# Pro-Inflammatory Protein PSCA Is Upregulated in Neurological Diseases and Targets β2-Subunit-Containing nAChRs

**DOI:** 10.3390/biom15101381

**Published:** 2025-09-28

**Authors:** Mikhail A. Shulepko, Yuqi Che, Alexander S. Paramonov, Milita V. Kocharovskaya, Dmitrii S. Kulbatskii, Anisia A. Ivanova, Anton O. Chugunov, Maxim L. Bychkov, Artem V. Kirichenko, Zakhar O. Shenkarev, Mikhail P. Kirpichnikov, Ekaterina N. Lyukmanova

**Affiliations:** 1 Faculty of Biology, Shenzhen MSU-BIT University, Shenzhen 518172, China; mikhail_shulepko@smbu.edu.cn (M.A.S.);; 2 Shemyakin-Ovchinnikov Institute of Bioorganic Chemistry, Russian Academy of Sciences, 117997 Moscow, Russia; apar@nmr.ru (A.S.P.); kocharovskaya.mv@phystech.edu (M.V.K.); d.kulbatskiy@gmail.com (D.S.K.); mlb@nmr.ru (M.L.B.); temakirich@nmr.ru (A.V.K.); zakhar.shenkarev@nmr.ru (Z.O.S.); info@mail.bio.msu.ru (M.P.K.); 3 Moscow Center for Advanced Studies, 123592 Moscow, Russia; ivanova.aa@phystech.edu; 4 Scientific Research Institute for Systems Biology and Medicine, 117246 Moscow, Russia; 5 Interdisciplinary Scientific and Educational School of Moscow University “Molecular Technologies of the Living Systems and Synthetic Biology”, Faculty of Biology, Lomonosov Moscow State University, 119234 Moscow, Russia

**Keywords:** prostate stem cell antigen, PSCA, Ly6/uPAR, nAChR, Alzheimer disease, aging, neurodegeneration, neuroinflammation

## Abstract

Prostate stem cell antigen (PSCA) is a Ly6/uPAR protein that targets neuronal nicotinic acetylcholine receptors (nAChRs). It exists in membrane-tethered and soluble forms, with the latter upregulated in Alzheimer’s disease. We hypothesize that PSCA may be linked to a wider spectrum of neurological diseases and could induce neuroinflammation. Indeed, PSCA expression is significantly upregulated in the brain of patients with multiple sclerosis, Huntington’s disease, Down syndrome, bipolar disorder, and HIV-associated dementia. To investigate PSCA’s structure, pharmacology, and inflammatory function, we produced a correctly folded water-soluble recombinant analog (ws-PSCA). In primary hippocampal neurons and astrocytes, ws-PSCA differently regulates secretion of inflammatory factors and adhesion molecules and induces pro-inflammatory responses by increasing TNFβ secretion. Heteronuclear NMR and ^15^N relaxation measurements reveal a classical β-structural three-finger fold with conformationally disordered loops II and III. Positive charge clustering on the molecular surface suggests the functional importance of ionic interactions by these loops. Electrophysiological studies in *Xenopus* oocytes point on ws-PSCA inhibition of α3β2-, high-, and low-sensitive variants of α4β2- (IC_50_ ~50, 27, and 15 μM, respectively) but not α4β4-nAChRs, suggesting targeting of the β2 subunit. Ensemble docking and molecular dynamics simulations predict PSCA binding to high-sensitive α4β2-nAChR at α4/β2 and β2/β2 interfaces. Complexes are stabilized by ionic and hydrogen bonds between PSCA’s loops II and III and the primary and complementary receptor subunits, including glycosyl groups. This study gives new structural and functional insights into PSCA’s interaction with molecular targets and provides clues to understand its role in the brain function and mental disorders.

## 1. Introduction

Neurological disorders represent one of the leading causes of global mortality and disability, whose prevalence has risen substantially over the past three decades, driven largely by demographic shifts including population growth and aging [1]. Among them, Alzheimer’s disease (AD), characterized by accumulation of β-amyloid peptide plaques in the brain, progressive memory loss, and cognitive decline, which all worsen with age, accounts for 60–70% of dementia cases. Currently, approximately 50 million people suffer from AD, and the number of people with AD is predicted to double within the next 25 years [2].

Pathological dysfunction of the cholinergic system of the brain is one of the basic mechanisms of the etiology of many neurological and mental disorders. The brain cholinergic system modulates synaptic plasticity [3,4] and mediates higher cognitive functions including attention [5], memory [6], and learning [7]. Cholinergic denervation has been recognized as a pathological hallmark of many neurodegenerative diseases including AD [8], Parkinson’s disease (PD) [9], and autistic spectrum disorders [10]. The key components of the cholinergic system are nicotinic acetylcholine receptors (nAChRs) of different subtypes, which are pentameric ligand-gated ion channels activated by the neurotransmitter acetylcholine [11,12]. These receptors play a crucial role in cognitive processes [13], addiction [14], and development of mental disorders [15]. The most abundant subtypes of nicotinic receptors in the human brain are heteromeric α4β2-nAChRs and homomeric α7-nAChRs [16], while other subtypes, such as heteromeric α3β2-nAChRs, show more restricted expression pattern [16]. The α4β2 and α7 receptors are susceptible to selective interaction with β-amyloid peptide, and their co-stimulation can reverse β-amyloid-induced synaptic dysfunction [17], driving interest in targeting of these ion channels for therapeutic intervention.

Three-finger or Ly6/uPAR proteins play important regulatory roles in many essential processes in the human body [18,19]. For example, the CD59 protein is involved in the control of the complement system [20], SLURP-1 and SLURP-2 control migration and proliferation of epithelial cells and protect them from oncogenic transformation [21,22], overexpression of Lypd6 and Lyd6b in the brain associates with autistic features [23], and Lynx1 is involved in the control of the cholinergic system in the brain [24,25]. Lynx1 [26], Lynx2 [27], Lypd6 [28], Lypd6b [29], Ly6H [30], Ly6g6e [31], and prostate stem cell antigen (PSCA) [32] are considered endogenous modulators of brain nAChRs. Some of these proteins, like Lynx1 and Lypd6, are well characterized [23,24], while others like PSCA are poorly studied. PSCA is moderately expressed in the human forebrain, brainstem, and cerebellum and choroid plexus cells [33]. It is also highly expressed in the telencephalon and peripheral ganglia of chickens and mice [32]. PSCA is bound to the cell membrane via a glycosylphosphatidylinositol anchor (GPI-anchor) [34], although a soluble form has also been detected in the human brain [35] and mouse cerebellum [36]. PSCA interacts with the α4 nAChR subunit, forming a stable complex, but no interaction occurs between PSCA and the α7 subunit [35]. In AD patients, the soluble PSCA level in the medial frontal gyrus is significantly elevated (by ~70%) compared to healthy donors [35]. In line with this, elevated expression of soluble PSCA has been observed in the cerebellum of 2xTg-AD mice [36], suggesting that dysregulation of PSCA expression or membrane localization may contribute to AD pathogenesis. PSCA expression has also been detected in brain tumors, such as medulloblastoma and glioma, implying a potential role in tumor progression [33]. Incubation of primary hippocampal neurons with soluble PSCA results in diminishing of the dendritic spine density [37]. Despite these findings indicating an interaction between PSCA and the α4-subunit-containing nAChRs, as well as the important role of PSCA in AD and cancer, the molecular and cellular mechanisms underlying its function in the brain remain unclear.

Here, we revealed that PSCA expression can be altered not only in AD and cancer, but also in various neurological disorders, and it can drive neuroinflammation. To explore the potential role of PSCA in the brain function, we produced a recombinant analog of soluble PSCA (ws-PSCA) and studied its spatial structure and interactions with heteromeric α4β2-, α3β2-, and α4β4-nAChRs. The data obtained provide new insights into PSCA’s molecular targets in the brain and establish a basis for further studies on its role in the development of various pathologies.

## 2. Materials and Methods

### 2.1. Bioinformatic Analysis

To analyze *PSCA* expression in different regions of the healthy brain, the GTEX database was used (dbGaP Accession phs000424.v10.p2 accessed 23 May 2025). The data, normalized as described in [38], were downloaded and analyzed using the GraphPad Prism 9.5.0 software (GraphPad Software, San Diego, CA, USA). Details of the statistical analysis are given in Table S1. To analyze age-related *PSCA* expression in the anterior cingulate cortex (ACC), the donors were divided into two age groups: 20–49 and 50–79 years.

For *PSCA* expression in different brain regions of patients with various nAChR-related neurodegenerative and neurological disorders, the data from the Gene Expression Omnibus database were analyzed using Geo2R. Analysis details are given in Figure S1 and Table S2.

### 2.2. Design of ws-PSCA Gene for Recombinant Production

The gene for recombinant production of water-soluble variant of human PSCA in *E. coli* was designed based on the amino acid sequence O43653 from the UniProt database. The codons were optimized according to *E. coli* codon usage frequency. The final gene sequence corresponds to the conserved Ly6 domain (Leu12-Ser86) of PSCA without the *N*-terminal signal peptide and the *C*-terminal sequence for attachment of the GPI-anchor, which are naturally present in premature PSCA. A start codon *ATG* encoding a methionine residue was added to the 5′ end. The gene was constructed from overlapping synthetic oligonucleotides using PCR and cloned into the *pET-22b(+)* vector.

### 2.3. Production and Characterization of Recombinant ws-PSCA

The *E. coli* expression strain BL21(DE3) was used for ws-PSCA production. Transformed cells were grown at 37 °C in TB medium. Protein expression was induced by addition of 0.05 mM isopropyl β-d-1-thiogalactopyranoside (IPTG) at A_600_ of 0.6. Extraction and purification of the target protein from cytoplasmic inclusion bodies were performed under denaturing conditions as described in [39]. Refolding was performed by buffer exchange of reduced ws-PSCA into a renaturation buffer (50 mM Tris/HCl, 1.5 M urea, 0.5 M L-arginine, 0.1 M NaCl, 3 mM GSH, 0.3 mM GSSG, pH 8.0) using the NAP-25 columns (Cytiva, MA, USA), dilution of ws-PSCA to the final concentration of 0.01 mg/mL, followed by incubation for 3 days at 4 °C and dialysis against ultrapure water. After renaturation, the protein was concentrated and purified using a reverse-phase C4 HPLC column (4.6 × 250 mm, A300, Jupiter, Phenomenex, Torrance, CA, USA).

For production of ^13^C,^15^N-labeled ws-PSCA for NMR studies, cells transformed with the *pET-22b(+)/PSCA* vector were grown in LB bacterial growth medium until the culture reached A_600_ of 0.6. Then, the cells were harvested (2000× *g* for 20 min) and transferred into a bioreactor (Sartorious, Goettingen, Germany) with M9 minimal medium supplemented by 10% of thiamine chloride and ^15^NH_4_Cl and ^13^C-glucose as sources of nitrogen and carbon, respectively. Afterward, gene expression was induced by the addition of 0.05 mM IPTG.

Protein purity, homogeneity, and correct folding were confirmed by HPLC, MALDI-MS, SDS-PAGE, and ^1^H-NMR spectroscopy. Protein concentration was determined spectrophotometrically according to ws-PSCA molecular mass (8350 Da) and molar extinction coefficient (10,595 M^−1^·cm^−1^) by measuring the UV absorbance at 280 nm.

### 2.4. Hippocampal Neurons and Astrocytes Isolation

Cultures of primary neurons and astrocytes were obtained as in [40,41], respectively. Wistar rats aged 8–20 weeks (Branch of IBCH RAS, Puschino, Russia) were mated for 1 week, after which the female spent the time remaining in her pregnancy in a separate cage. Animals were bred randomly, without the use of randomization tools. Eight rat pairs were mated for the experiments. After birth, the pups were taken away within 24 h. A total of 8 Rat pairs were mated for the experiments. The sex of the pups was not determined, and investigation was conducted using the neurons and astrocytes isolated from the hippocampi of pups of both sexes. The animals were kept in standard conditions of the Laboratory Animal Nursery of the IBCH RAS, having the international accreditation AAALACi. No randomization was performed. There were no specific inclusion/exclusion criteria for animals in the experiment.

To obtain a culture of primary neurons, newborn rat pups were anesthetized, decapitated, and the hippocampus was isolated, homogenized with a scalpel, and incubated for 15 min in a 0.8% trypsin solution in DMEM (PanEco, Moscow, Russia). Then the hippocampal homogenate was centrifuged at 500× *g* for 2 min. The sediment was dissolved in 7 mL of Neurobasal-A medium (Gibco, Waltham, CA, USA) supplemented with NeuroMax additive (PanEco) and pipetted until an opalescent homogeneous mass was formed. Neurons were then seeded onto poly(L)-lysine-coated glass (PanEco) in 24-well plates (SPL Lifesciences, Pocheon, Korea; 1 × 10^5^ cells per well). After 1 h, the medium was replaced by a fresh one. On the third day, 20 μM cytarabine (Sigma-Aldrich, St. Louis, MO, USA) was added to the medium to inhibit the growth of glial cells. Neurons were cultured for additional 11 days with medium changes every 4 days.

To obtain a culture of primary astrocytes, the opalescent homogenous mass obtained as described above was seeded into poly(L)-lysine-coated 6-well plates (SPL Lifesciences) and after 1 h Neurobasal-A medium was replaced with DMEM/F12 medium (PanEco) supplemented with G-5 additive (PanEco). Astrocytes were cultured for 42 days with sub-culturing twice per week, and cells were detached by Versene solution (PanEco). After 42 days of culturing, GFAP content was analyzed by flow cytometry.

On the 14th day, 1 μM of ws-PSCA from 1 mM stock solution in 100% DMSO (the same concentration as was used for ws-Lynx1 in the previous work [37]) was added to the neurons in 24-well plates (1 × 10^5^ cells), and neurons were additionally incubated for 7 days. On the 42nd day, astrocytes were seeded in 6-well plates (1 × 10^5^ cells) and incubated with 1 μM ws-PSCA from 1 mM stock solution in 100% DMSO for additional 7 days. To check the possible DMSO effect, the neurons and astrocytes incubated with an equal amount of DMSO were used.

### 2.5. Analysis of Inflammatory Cytokines Secretion by Neurons and Astrocytes

To study the effect of ws-PSCA on the secretion by the neurons and astrocytes of cytokines and intercellular adhesion molecules involved in synaptic function, the LegendPlex immunoassay kit (740946, BioLegend, San Diego, CA, USA) was used. Media (25 μL) were collected from neurons and astrocytes treated with ws-PSCA or DMSO, immunoassayed according to the manufacturer’s protocol, and analyzed using the Attune NxT flow cytometer (Life Technologies, Carlsbad, CA, USA) and the Attune NxT Software 2.3. (Life Technologies). CD44 and NCAM were excluded from the analysis due to degradation of protein standards. The levels of secreted TNFα, TNFβ, IL10, and IL12 p40 were assayed by ELISA according to manufacturer’s instructions. The following kits were used: KHC3011 (Invitrogen, Waltham, CA, USA) for TNFα, BMS202 (Invitrogen) for TNFβ, BMS215/2 (eBioscience, Waltham, CA, USA) for IL10, and KHC0121 (Invitrogen) for IL12 p40. To determine the concentrations of substances, a calibration curve (5-parameter non-linear regression) in the GraphPad Prism 9.5.0. software was used. Interpolation curves for immunoassays and gating strategy for the LegendPlex immunoassay are shown in Figure S2 and Figure S3, respectively.

### 2.6. Study of Structure and Dynamics by NMR Spectroscopy

For NMR studies, ^13^C,^15^N-labeled and non-labeled ws-PSCA samples were used. The NMR samples were prepared by dissolving lyophilized proteins in 0.3 or 0.5 mL of deionized water, respectively. D_2_O (5%) was added to the samples, and pH was adjusted to 7.0 by concentrated HCl or NaOH. The samples were placed into 5 mm NMR sample tubes (Shigemi tubes were used for the ^13^C,^15^N-labeled sample). The final ws-PSCA concentrations were 0.07 mM and 0.17 mM for the ^13^C,^15^N-labeled, and non-labeled samples, respectively. To transfer the protein into D_2_O solution, NMR samples were lyophilized and dissolved in D_2_O (100% ^2^H).

The NMR spectra were measured using the AVANCE-III-600 and AVANCE-III-800 spectrometers (Bruker, Billerica, MA, USA) equipped by CryoProbes. All spectra were measured at a temperature of 37 °C. Three-dimensional spectra were acquired using a non-uniform sampling method with 30% of sparse sampling for triple-resonance (^1^H,^13^C, and ^15^N) experiments and 50% for 3D TOCSY-HSQC and NOESY-HSQC spectra, and were processed by MDDNMR [42]. Using acquired standard set of 3D triple-resonance NMR experiments: HNCO, HNCA, HNCACB, HN(CO)CA, HN(CO)CACB, and HN(CA)CO backbone resonance assignment were performed. Three-dimensional ^13^C-HCCH-TOCSY [43], ^15^N-filtered 3D TOCSY-HSQC (t_m_ of 80 ms), and NOESY-HSQC (t_m_ of 120 ms) spectra were used for side chains assignment. Using the non-labeled ws-PSCA sample, 2D ^1^H-^1^H NOESY (t_m_ of 100 ms) and TOCSY (t_m_ of 80 ms) spectra were acquired in both H_2_O and D_2_O solutions. ^3^J_H_^N^_H_^α^ and ^3^J_NH_^β^ scalar coupling constants were measured using the 3D HNHA spectrum and qualitatively estimated using the 3D HNHB spectrum, respectively [44]. Temperature gradients of amide protons (Δδ^1^H^N^/ΔT) were extracted from a series of ^15^N-HSQC spectra measured in the 20–45 °C temperature range with 5 °C steps. The H/D exchange kinetics was measured using ^15^N-HSQC spectra obtained immediately after dissolving of lyophilized ^13^C,^15^N-labeled ws-PSCA in D_2_O.

The relaxation parameters of ^15^N nuclei (longitudinal (R_1_) and transverse (R_2_) relaxation rates and steady-state heteronuclear ^15^N-{^1^H} NOEs) were measured for the ^13^C,^15^N-labeled protein at 37 °C and 81 MHz. Relaxation measurements were performed using a standard set of ^15^N-HSQC-based pseudo 3D experiments [45].

Resonance assignment of ^1^H, ^13^C, and ^15^N nuclei was performed using the obtained 2D and 3D NMR spectra [46] via the CARA 1.8 software (Keller and Wüthrich, ETH, Zurich, Switzerland). The secondary structure of ws-PSCA was calculated from the determined chemical shifts using TALOS-N 4.21 [47]. Distance constraints for the 3D structure calculation were derived from cross-peak intensities in ^15^N-filtered 3D NOESY-HSQC (t_m_ of 120 ms) and 2D ^1^H-^1^H NOESY (t_m_ of 100 ms) spectra. The φ and χ1 dihedral angles restraints were obtained from J-couplings, NOE, and TALOS-N data. For application of the hydrogen bonding restraints, amide protons demonstrating that Δδ^1^H^N^/ΔT > −4.5 ppb/K and half-exchange ^1^H/^2^H time > 20 min were considered to be hydrogen bond donors. Additional distance restraints were applied to hold disulfide connectivity. Three-dimensional structures were calculated using CYANA ver. 3.98 [48]. 400 structures were calculated and 20 structures with minimal target function were selected for analysis. Visualization and analysis of the calculated structures were performed using MOLMOL ver. 2K.2 [49]. The ^15^N relaxation data were analyzed in terms of the model-free approach using the ModelFree 4.15 software [50] together with FastModelFree software 1.01 [51]. The isotropic model for overall diffusion was used.

### 2.7. Accession Codes

Experimental restraints, chemical shifts, and the calculated 3D structure of ws-PSCA were deposited into the PDB (9U9N) and BMRB (36748) databases.

### 2.8. Electrophysiology Recordings in X. laevis Oocytes

Stage V-VI oocytes surgically isolated from anesthetized *Xenopus laevis* (RRID: NXR_0.0080) were defolliculated by 60–90 min enzymatic treatment with 1.5 mg/mL collagenase Type IA in Ca^2+^-free ND96 solution (96 NaCl, 2 KCl, 1.8 CaCl_2_, 2 MgCl_2_, 5 mM HEPES, 50 μg/mL gentamycin, pH 7.4) was followed by the microinjection of 20 nL mRNA mixtures containing human nAChR subunit transcripts (α3, α4, β2, β4) at specified α:β molar ratios: 1:1 (0.55:0.45 μg/μL) for α3β2- and α4β4-nAChRs, and 10:1 or 1:10 (0.9:0.09 or 0.09:0.9 μg/μL) for (α4)_3_(β2)_2_ (low-sensitive, LS) and (α4)_2_(β2)_3_ (high-sensitive, HS) nAChR variants, respectively. The mRNA was synthesized using the T7 mMessage mMachine kit (Thermo Fisher Scientific, Waltham, MA, USA) with a total mRNA concentration of 1 mg/mL. Post-injection oocytes were incubated at 18 °C in ND96 medium for 24–72 h prior to two-electrode voltage-clamp recordings using the TEC-03X amplifier (NPI electronic GmbH, Tamm, Germany) at −50 mV holding potential, with 5 s acetylcholine (ACh) applications interspersed by 5 min ND96 washouts to attenuate desensitization, following established signal acquisition and analysis protocols [52]. The ACh concentration was 10 μM for HS α4β2-nAChR and 100 μM for LS α4β2-, α3β2-, and α4β4-nAChRs. Voltage and current electrodes were filled with 3 M KCl. The resistances of both electrodes were kept between 0.7 and 1.5 MΩ. Current amplitudes were quantified using ClampFit 10.7 (baseline-to-peak analysis (Molecular Devices, San Jose, CA, USA)), and dose–response curves were fitted by the Hill equation in GraphPad Prism 9.5.0.

### 2.9. Statistical Analysis

Data are presented as mean ± SEM. Specific sample sizes (n) and statistical methodologies are detailed in corresponding figure legends. Before the comparisons, the data were tested for normality (Shapiro–Wilk test, at *p* = 0.05). The data were analyzed using one sample *t*-test followed by Holm–Sidak’s post hoc test for normally distributed data. Two-sided Mann–Whitney u-test or Kruskal–Wallis test followed by a post hoc Dunn’s test were used for data with non-Gaussian distribution as indicated in the figure legends. The difference between the data groups was considered statistically significant at *p* < 0.05. The “*” symbol was used to designate the normally distributed data, while the “#” symbol was used to show the data with non-Gaussian distribution. Analysis was performed using the GraphPad Prism 9.5.0 software. The group size was determined according to previous studies [27,28,52].

### 2.10. Computer Modeling of HS and LS α4β2-nAChR Stoichiometries

The overall modeling pipeline is provided in Figure S4.

To model HS and LS stoichiometries of α4β2-nAChR, cryo-EM structures of the receptor with PDB codes 8ST1 and 8SSZ, respectively, were used as templates [53]. Missing structured regions were rebuilt using AlphaFold DB [54] predictions (accessed via the UniProt identifiers P43681 for the α4 subunit and P17787 for the β2 subunit). In both models, the M4 helix was reconstructed for the β2 subunit. For the LS α4β2-nAChR model, structured intracellular segments were added. Short unstructured regions near the *N*- and *C*-termini were modeled using the MODELLER 9.19 software [55]. Most of the intracellular domain was not included in the model due to unreliable quality as indicated by low predicted confidence scores in AlphaFold DB (for details, see Figure S4, *steps 1b*, *1c*). The obtained models were N-glycosylated at the N29, N79, and N146 residues of the α4 subunit and N26, N143, and N460 of the β2 subunit. The glycosylated forms of α4β2-nAChR were used for MD calculations, while for subsequent docking glycosyl moieties were removed.

### 2.11. Molecular Dynamics Simulations

All molecular dynamics (MD) calculations were performed using GROMACS 2024.4 [56] with the CHARMM36m force field [57]. Before modeling, the receptors were immersed into a lipid bilayer (composition: dioleoylphosphatidylethanolamine (DOPE)/dioleoylphosphatidylcholine (DOPC)/*N*-palmitoyl-D-sphingomyelin (PSM)/cholesterol at the 2:1:5:2 ratio) and solvated with TIP3P water molecules using the CHARMM-GUI webserver [58]. There were two types of MD simulations:

1. The first type of MD simulations served for conformational sampling of isolated ws-PSCA and nAChRs to generate unlike conformations as an input for the ensemble docking (Figure S4, *steps 1–3*):
  - For PSCA, four NMR-derived representative conformations were selected and each were subjected to 200 ns MD simulations in aqueous solution; resulting aggregated 800 ns trajectory was clustered using the *gmx cluster* utility and *gromos* clustering method, with a cutoff value of 0.250 nm, yielding 93 clusters of conformations (Figure S4, *steps 1–3a*).
  - For nAChRs, two 600 ns MD simulations for HS and LS stoichiometries were calculated (Figure S4, *steps 2b*, *2c*), from which individual dimeric interaction interfaces were extracted and concatenated: β2(+)/β2(−) (600 ns), α4(+)/β2(−) (2400 ns), and β2(+)/α4(−) (2400 ns); this was done using the *gmx trjconv* and *gmx trjcat* utilities. These trajectories were clustered using the following cutoff values: 0.140 nm (β2(+)/β2(−); 53 clusters), 0.150 nm (α4(+)/β2(−); 54 clusters), and 0.172 nm (β2(+)/α4(−); 57 clusters) (Figure S4, *steps 3b–d*). Clustering was performed using the defined binding site residues (see Section 2.12 below), excluding the M4 helix terminus (residues 455–477) of the β2-subunit due to its high flexibility.
2. Secondly, two MD replicas (500 ns) were aimed to assess the stability of the proposed model of the HS (α4)_2_(β2)_3_-nAChR complex with ws-PSCA after the ensemble docking and to characterize intermolecular contacts (Figure S4, *step 7*). An in-house Impulse software [59] was used for the latter, omitting the first 30 ns of each trajectory estimated to equilibrate the system.

### 2.12. Ensemble Docking of the nAChR/PSCA Complex

To account for the conformational flexibility of interacting molecules, we employed the so-called ensemble docking protocol (see Figure S4), similar to our previous work [60]. Sets of MD-derived conformations were used as input for the MEGADOCK program [61]. To avoid implausible docking poses, only the canonical binding site at nAChR subunits was allowed for docking, including the following residues (colored in Figure S4, *step 3b–3d*):

- β2(+): 21–35, 46–50, 60–64, 94–103, 110–120, 129–167, 177–206, 261–271, and 455–477;
- β2(−): 31–45, 52–63, 76–83, 106–125, 136–142, 158–184, 204–210, and 455–477;
- α4(+): 24–38, 49–53, 62–67, 97–105, 113–123, 132–172, 182–214, and 267–277;
- α4(−): 33–49, 54–66, 77–87, 109–128, 139–146, 161–187, and 210–216.

For each of the 93 × 53 = 4929 (β2(+)/β2(−) interface), 93 × 54 = 5022 (α4(+)/β2(−) interface), and 93 × 57 = 5301 (β2(+)/α4(−) interface) docking runs, MEGADOCK systematically generated 3600 solutions, and the 100 top-scoring solutions were used for further analysis (Figure S4, *step 4*). The obtained 492,900, 502,200, and 530,100 solutions, respectively, were post-scored in two stages.

The first-stage (“non-specific”, Figure S4, *step 5*) discarded solutions that failed to meet the following criteria (estimated from the analysis of the docking ensembles (Figure S5) and previously shown to be favorable for protein–protein docking tasks [60]):

- Buried surface area ≥ 2750 Å^2^;
- Molecular hydrophobic potential’s [62] complementarity score ≥ 0.5;
- Number of intermolecular ionic bonds ≥ 6;
- Number of intermolecular hydrogen bonds ≥ 7.

These parameters were calculated using the PLATINUM software package [62]. The “non-specific” post-scoring stage reduced the number of solutions to 5483, 4075, and 1671 for the β2(+)/β2(−), α4(+)/β2(−), and β2(+)/α4(−) interfaces, respectively.

The second (“specific”) post-scoring stage was based on the frequency of intermolecular contacts observed in the docking ensembles (Figure S4, *step 6* and Figure S6). Since the β2(+)/α4(−) interface exhibited a decreased ability to interact with the ligand (see Figures S5 and S6), just two remaining interfaces (β2(+)/β2(−) and α4(+)/β2(−)) were kept for further analysis, producing the following list of the intermolecular receptor/ligand interactions imposed to occur in the α4β2-nAChR/ws-PSCA complexes:

**at the β2(+)/β2(−) interface,**
  - K147(+), R186(+) → E13, D14, D52, D53, D68
  - D192(+), D193(+) → R32, R34
  - D170(−) → R32, R34
  - D171(−) → R32, R34
**at the α4(+)/β2(−) interface,**
  - R193(+), K194(+) → E13, D14, D52, D53, D68
  - D170(−) → R32, R34
  - D171(−), E196(+) → R32, R34

This stringent filtering yielded only 14 solutions for the β2(+)/β2(−) interface and 19 solutions for α4(+)/β2(−) interface. Finally, the best solution for each interface was chosen through visual inspection (Figure S6, *right panels*). These solutions were used to reconstruct the full HS (α4)_2_(β2)_3_-nAChR/ws-PSCA complex model, including the whole pentameric receptor, which was further subjected to two MD replicas (500 ns each, see above).

## 3. Results

### 3.1. PSCA Expression in the Human Brain

**Figure 1:**
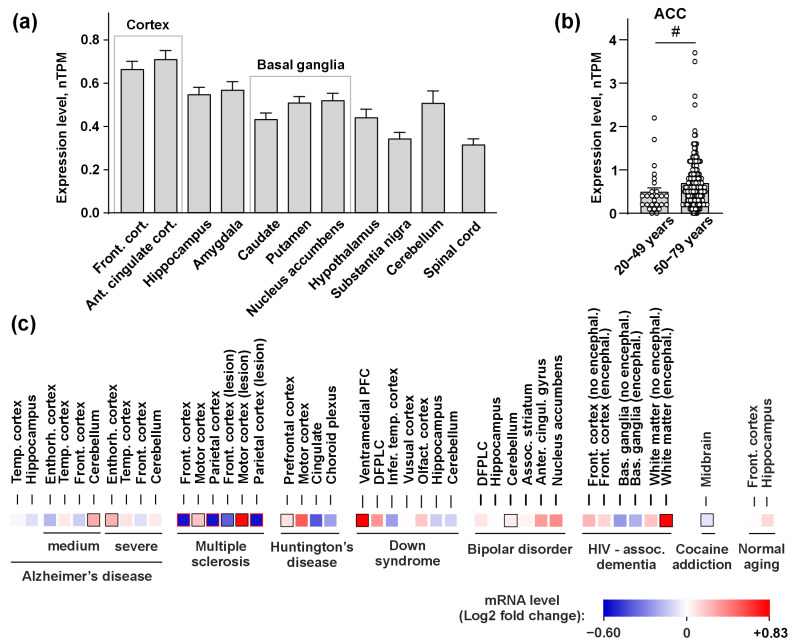
*PSCA* expression in the brain of healthy individuals and in various pathologies. (**a**) *PSCA* expression in the healthy brain in accordance with the GTEX database. Data represent a normalized number of transcripts per million ± SEM (n = 139–246). The details of Kruskal–Wallis test followed by Dunn’s post hoc test are presented in Table S1. (**b**) *PSCA* expression in the ACC of healthy individuals aged 20–49 and 50–79 years in accordance with the GTEX database. Data represent the normalized number of transcripts per million ± SEM (n = 26–183), # (*p* < 0.05) indicates significant difference between the data groups according to the two-sided Mann–Whitney u-test. (**c**) *PSCA* expression in the brain of patients with neurological and neuropsychiatric disorders in accordance with the Gene Expression Omnibus database are analyzed by Geo2R. Significant changes in comparison with donors without designated diseases by two-sided Mann–Whitney u-test are shown by black/red frames. The dataset accession numbers, number of patients, and statistical details are given in Figure S1 and Table S2.

Previously, it was found that PSCA protein expression is increased by ~70% in the medial frontal gyrus of patients with AD [35]. Here, we evaluated a *PSCA* mRNA expression in the healthy brain and in different neurological and mental diseases associated with the cholinergic system dysfunction. Analysis of the GTEX database revealed the *PSCA* expression in the cerebral cortex, hippocampus, amygdala, basal ganglia, hypothalamus, substantia nigra, and cerebellum of the healthy brain, as well as in the spinal cord (Figure 1a, Table S1). Additionally, we analyzed changes in the *PSCA* expression during aging and found that its level is increased in the ACC of humans aged ≥ 50 years (Figure 1b). No age-related changes were observed in other brain regions.

Some neurodegenerative disorders, such as AD, PD, and multiple sclerosis (MS) are associated with aging [63]. Indeed, increased *PSCA* levels were revealed in the entorhinal cortex of patients with severe stage of AD and in the cerebellum of patients with medium stage of AD. Additionally, we found significantly increased levels of *PSCA* in the motor cortex of the patients with MS, the prefrontal cortex of the patients with Huntington’s disease, the ventromedial prefrontal cortex of the patients with Down syndrome, in the cerebellum of the patients with bipolar disorder, the white matter of the patients with HIV and dementia, and in the midbrain of cocaine-addicted individuals (Figure 1c). However, *PSCA* was reduced in the frontal and parietal cortex of the patients with MS compared with healthy individuals (Figure 1c). These data suggest implications of PSCA in the development of various brain pathologies and aging.

### 3.2. Bacterial Production of Recombinant ws-PSCA

**Figure 2:**
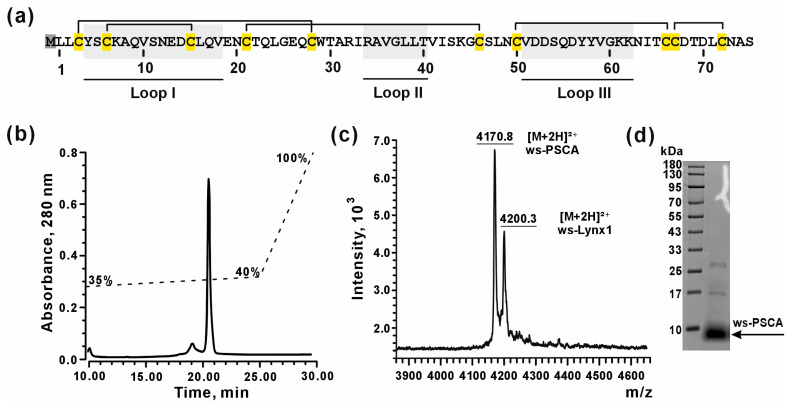
Characterization of ws-PSCA. (**a**) Amino acid sequence of ws-PSCA. Cysteine residues are shown in yellow, disulfide bonds are indicated by lines, and the protein sequence corresponding to the loop regions is highlighted by the gray background. (**b**) Representative HPLC chromatogram of purified ws-PSCA. (**c**) MALDI-MS spectrum of the refolded ws-PSCA (expected average *m*/*z* of the [M+2H]^2+^ ion: 4171.2 Da). Ws-Lynx1 was used as a reference protein (expected average *m*/*z* of the [M+2H]^2+^ ion: 4200.8 Da). (**d**) SDS-PAGE analysis of the refolded ws-PSCA (MW ~ 8.3 kDa). The uncropped and unedited original image of the SDS-PAGE gel is given in Figure S7.

Human PSCA cannot be purified from a natural source. Thus, to perform structural and functional studies of PSCA, we developed an *Escherichia coli* expression system for recombinant production of water-soluble variant of PSCA (ws-PSCA) lacking the *N*-terminal signal peptide sequence and *C*-terminal sequence for GPI-anchoring to the cell membrane. PSCA contains five disulfide bonds (Figure 2a); therefore, to produce correctly folded ws-PSCA, we used a protocol for protein expression in the form of cytoplasmic inclusion bodies, followed by purification under denatured conditions and further refolding. This approach has been successfully used by us for production of several Ly6/uPAR proteins [64,65,66,67]. The final yield of refolded ws-PSCA and its ^13^C,^15^N-labeled analog was ~2.5 and ~1 mg per 1 L of bacterial culture, respectively. Due to the starting codon required for translation, recombinant ws-PSCA contained the additional *N*-terminal methionine residue (Met0, Figure 2a). The homogeneity and purity of refolded ws-PSCA was confirmed by HPLC, mass spectrometry, and SDS-PAGE analysis (Figure 2b–d). The MALDI-MS analysis confirmed the formation of 5 disulfide bonds in the molecule of ws-PSCA. The observed average *m*/*z* value (4170.8 Da) of the [M+2H]^2+^ ion corresponded to the theoretically calculated (4171.2 Da) value for correctly folded ws-PSCA protein (Figure 2c).

### 3.3. Ws-PSCA Regulates Secretion of Inflammatory Factors and Adhesion Molecules by Neurons and Astrocytes

**Figure 3:**
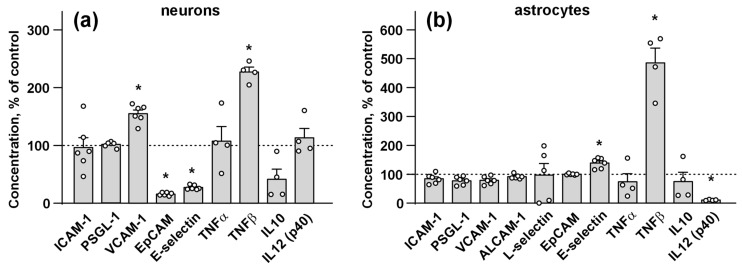
Influence of ws-PSCA on the secretion of inflammatory factors and adhesion molecules by the neurons (**a**) and astrocytes (**b**). Data represent concentrations in the culture media normalized to the control (untreated cells, 100%, dashed line) ± SEM (n = 4–6). * (*p* < 0.05) indicates the significant difference between the data groups by one sample *t*-test with Holm–Sidak’s post hoc test.

To investigate the possible role of PSCA in neuroinflammation, which is often observed in neurological diseases [68,69,70,71,72], we assayed the influence of ws-PSCA on secretion of various inflammatory factors (ALCAM-1, L-selectin, TNFα, TNFβ, IL10, and IL12 p 40) and adhesion molecules (ICAM-1, PSGL-1, VCAM-1, EpCAM, E-selectin) by the primary neurons and astrocytes. Analysis by flow cytometry and ELISA revealed that incubation of the neurons with ws-PSCA increased the secretion pro-inflammatory adhesion factor VCAM-1 and pro-inflammatory cytokine TNFβ and decreased secretion of adhesion factors EpCAM and E-selectin (Figure 3a). For the astrocytes, incubation with ws-PSCA upregulated secretion of E-selectin and TNFβ (Figure 3b); however, secretion of pro-inflammatory cytokine IL12 p40 was significantly diminished (Figure 3b). Notably, in the neurons, ws-PSCA did not affect the secretion of inflammation stimulator ICAM-1, leukocyte migration regulator PSGL-1, pro-inflammatory cytokine TNFα, and immunosuppressive IL10. In the astrocytes, ws-PSCA did not alter the secretion of ICAM-1, PSGL-1, VCAM-1, EpCAM, TNFα, IL10, ALCAM-1, and L-selectin. Neither the astrocytes nor the neurons secreted ICAM-2, ICAM-3, Pecam, and P-selectin, and the neurons also did not secrete ALCAM-1 and L-selectin (for cytokines, which were not secreted by cells, the data was not shown because concentrations were calculated as “zero”, the interpolation curves of protein standards are in Figure S2).

### 3.4. NMR Structure and Dynamics of ws-PSCA in Aqueous Solution

**Figure 4:**
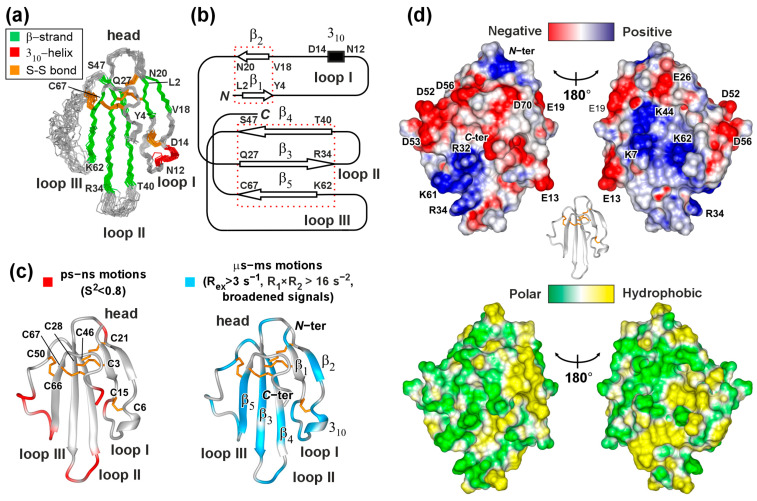
NMR structure and dynamics of ws-PSCA (submitted to PDB under the 9U9N code). (**a**) Set of the 20 best CYANA structures of ws-PSCA. Protein backbone and disulfide bonds (*orange*) are shown. Secondary structure elements are color-coded: *red*—3_10_-helix, *green*—β-sheet. (**b**) Scheme of the secondary structure elements. β-sheets formed by the β1/β2 and β3/β4/β5 strands are highlighted by *red dotted rectangles*. (**c**) Ribbon representation of the ws-PSCA structure with mapped “fast” (ps–ns timescale, *left*) and “slow” (µs-ms timescale, *right*) backbone dynamics. (*Left*) High-amplitude ps–ns mobility was observed in the regions with order parameter S^2^ < 0.8 (*red*). (*Right*) Significant µs-ms conformational fluctuations were detected for the residues, in which the contribution of exchange to the R_2_ relaxation rate R_ex_ > 3 s^−1^ (800 MHz), the product of R_1_ × R_2_ > 16 s^−2^ [74], or a significant broadening of the HN signal was observed, including the signals, which were not observed in the spectra (*blue*). (**d**) Two-sided views of the ws-PSCA molecular surface with distribution of electrostatic potential (*top*) and molecular hydrophobicity potential [62] (*bottom*).

Using the set of 3D NMR spectra, nearly complete ^1^H, ^13^C, and ^15^N assignment of the main chain and side chains of the ws-PSCA molecule was obtained following a standard procedure (Figure S8). The resonances of Leu1, Ala31, and Gly45 main chain amide groups were significantly broadened, probably due to exchange processes. The resulting chemical shifts were used to calculate the secondary structure of ws-PSCA using the TALOS-N program [73]. The data revealed the predominant β-structure of ws-PSCA containing five β-strands (Figure S9). The spatial structure of ws-PSCA was calculated in the CYANA program using NMR-based constraints on interproton distances, torsion angles φ and χ1, and hydrogen bonds, as well as standard restraints to support the disulfide bond connectivity. The final set of 20 structures is shown in Figure 4a, and structural statistics are presented in Table S3. Ws-PSCA demonstrated a characteristic β-structural fold with three loops (fingers) (Figure 4a,b). The first loop reveals a short β-sheet formed by two β-strands (Leu2–Tyr4 and Val18–Asn20) and a single turn of 3_10_-helix. Residues from loops II and III formed a wider and longer β-sheet containing three β-strands: Gln27–Arg34 and Thr40–Ser47 from loop II and Lys62–Cys67 from loop III. The ws-PSCA molecule is stabilized by a common β-sheet network of hydrogen bonds (Figure S10) and by several additional hydrogen bonds between the backbone of *N*-terminal residues and the side chains of the *C*-terminal fragment (H^N^ Leu2—O^δ^ Asp70, H^N^ Tyr4—O^δ^ Asn73, and H^δ2^ Asn73–OC’ Tyr4).

Good structural convergence was observed in the β-structural regions of ws-PSCA (backbone Root Mean Square Deviation (RMSD) value over 20 NMR structures of the Leu1-Ile33, Val41-Cys50, and Asn63-Ala74 regions was ~0.71Å, Table S3), while the tip of loop II (Arg34–Thr40) and lateral part of loop III (Val51–Lys62) were disordered (Figure 4a). ^15^N relaxation measurements confirmed high-amplitude intramolecular dynamics of the protein backbone in these regions. Some of the residues located in the disordered regions demonstrated reduced values of the order parameter S^2^ (less than 0.8), indicating increased mobility on a “fast” time scale (ps-ns, Figure 4c, *left*). Other regions of the ws-PSCA backbone were stable in this time scale (Figure 4c and Figure S10). At the same time, a large number of the ws-PSCA residues were involved in “slow” dynamics (μs-ms time scale, Figure 4c, *right*), as it was identified by significant exchange contributions to R_2_ relaxation rates (R_ex_ > 3 s^−1^), increased values of R_1_×R_2_ product (>16 s^−2^), or broadening of the HN signals (Figures S11 and S12). Broadening was qualitatively estimated from the cross-peaks intensity in the 3D HNCO spectrum. The residue was considered broadened if its HNCO cross-peak was invisible or its intensity was five times less than the average signal intensity in the “stable” regions of the ws-PSCA backbone (where R_ex_ < 3 s^−1^ and S^2^ > 0.8: Leu2-Ser5, Asp68, Thr69, Leu71, Figure S12). Almost all regions of ws-PSCA exhibited significant mobility in the μs-ms time scale (Figure 4c, *right*).

From the calculated distribution of an electrostatic potential at the ws-PSCA surface, the clustering of the positively charged residues became evident. One cluster was formed by the residues of loops II and III (Arg32, Arg34, Lys61), while the second cluster was formed on the opposite side of the molecule by the Lys4, Lys43, and Lys62 residues (Figure 4d). Negatively charged residues showed more uniform distribution. According to the calculated molecular hydrophobicity potential, hydrophobic and polar groups on the ws-PSCA surface did not show pronounced clustering (Figure 4d).

### 3.5. Ws-PSCA Inhibits α4β2- and α3β2-nAChRs, but Not α4β4-nAChRs

**Figure 5:**
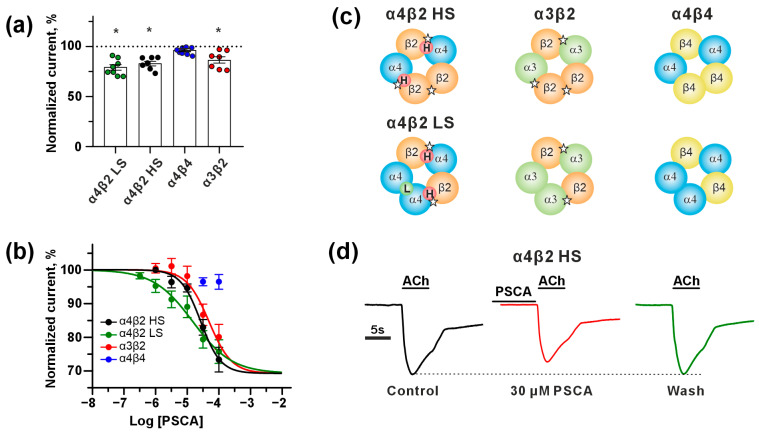
Characterization of ws-PSCA action at various nAChR subtypes. (**a**) Effect of 30 μM ws-PSCA on ACh-evoked currents at HS/LS α4β2-, α4β4-, and α3β2-nAChRs. Normalized ACh-evoked currents (relative to the control in absence of ws-PSCA, dashed line at 100%) are shown as mean ± SEM (n = 7–8 oocytes from ≥ 3 frogs). * (*p* < 0.05) indicates statistical difference from the control by one-sample *t*-test with the Holm–Sidak post hoc test. (**b**) Dose–response curves for inhibition of ACh-evoked currents at (HS/LS) α4β2- and α3β2-nAChRs by ws-PSCA. The effect of 30 and 100 µM ws-PSCA on α4β4-nAChRs is also shown. The data are normalized to the control (100%), presented as mean ± SEM (n = 5–8 oocytes from ≥ 3 frogs), and fitted by the Hill’s equation (see parameters in Table 1). (**c**) Stoichiometry of nAChR subtypes. High- and low-sensitive binding sites at α4β2-nAChRs are shown by red and green circles, respectively. The sites of possible ws-PSCA bindings are shown by stars. (**d**) Average ACh-evoked current traces for HS α4β2-nAChRs in the absence (black for the control, green for wash-out) or presence (red) of 30 μM ws-PSCA (n = 7 oocytes from ≥ 3 frogs). Pulses of 10 μM ACh lasting 5 s were used (shown by bars). Oocytes were pre-incubated with ws-PSCA for 20 s (bars drawn out of scale).

Previous studies have demonstrated the binding of recombinant PSCA to the α4 nAChR subunit extracted from the human temporal cortex homogenate, but not to the α7 nAChR subunit [35], although no further studies of PSCA action on α4-subunit-containing nAChRs were performed. Here, we studied for the first time a pharmacology of ws-PSCA at different heteromeric nAChR subtypes containing α4 subunits: high-sensitive (HS) (α4)_2_(β2)_3_, low-sensitive (LS) (α4)_3_(β2)_2_, and α4β4 expressed in *X. laevis* oocytes. The nicotinic receptor of the α3β2 subtype was used as a control. We found that ws-PSCA at the 30 μM concentration reversibly inhibited ACh-evoked currents at all studied nAChRs except α4β4 receptor subtype (Figure 5a). Ws-PSCA showed no effect on α4β4-nAChR at concentrations up to 100 μM (Figure 5b and Figure S13), suggesting a selective mechanism of action on α3β2- and α4β2-nAChRs.

The inhibition of ACh-evoked currents through α4β2/α3β2-nAChRs by ws-PSCA was dose-dependent with IC_50_ values of 15 ÷ 50 μM and a maximal reduction in the current amplitude of ~30% relative to the control (Figure 5b and Table 1). The maximum achievable concentration of ws-PSCA (100 μM) was insufficient to precisely define the levels of maximal inhibition (*bottom* parameters) for α3β2- and LS α4β2-nAChRs, so all three datasets were fitted simultaneously using a single level of maximal inhibition. For LS α4β2-nAChR, we observed a highly variable inhibitory effect of ws-PSCA with a gentle Hill slope, which may be related to the reduced cooperativity due to the ws-PSCA binding to the LS site or to the variability in the expression level of the β2 subunit against the background excess of the α4 subunit, resulting in significant variability of the binding site concentration.

The observation that ws-PSCA did not affect the currents through α4β4-nAChRs, but inhibited β2-subunit-containing α4β2- and α3β2-nAChRs, indicates that the β2 subunit is a possible target of this protein. Indeed, the comparison of the stoichiometries of the tested receptors suggests that ws-PSCA can interact with the α4/β2 and α3/β2 interfaces and possibly with the β2/β2 and β2/α4 interfaces, but not with the α4/α4 and α4/β4 sites (Figure 5c). Despite the fact that the inhibitory effect was higher for the α4β2 receptors, the difference with α3β2-nAChRs did not reach significance (Figure 5, Table 1). Notably, the application of ws-PSCA alone did not elicit the currents at the tested receptors, and the observed inhibition was completely reversible (Figure 5d).

### 3.6. Computer Modeling of the α4β2-nAChR/ws-PSCA Complex

To understand the possible molecular basis of the ws-PSCA action on α4β2-nAChRs, we modeled the ws-PSCA binding to this receptor taking into account its two possible stoichiometries (HS: (α4)_2_(β2)_3_ and LS: (α4)_3_(β2)_2_). For this purpose, we performed a so-called ensemble docking with post-scoring [60]. A detailed concept of computer modeling is described in Section 2 and is illustrated by Figure S4.

To initialize ensemble docking, MDs of isolated ws-PSCA in water solution and HS and LS α4β2-nAChRs in a mixed membrane (DOPE/DOPC/PSM/cholesterol = 2:1:5:2) were performed separately (Figure S4, *steps 1a*,*b*,*c–2a*,*b*,*c*). Resulting trajectories were conformationally clustered to produce 93 conformations of PSCA (Figure S4, *step 3a*) and several dozen conformations of possible binding sites located at the interfaces between pairs of primary (+) and complementary (−) nAChR subunits. For α4(+)/β2(−), β2(+)/α4(−), and β2(+)/β2(−) interfaces, 54, 57, and 53 conformations were produced, respectively (Figure S4, *step 3b–d*). The α4(+)/α4(−) interface was not analyzed as its targeting contradicts the electrophysiology data (Figure 5c). Combinatorial protein–protein docking produced 100 top-scoring solutions in each elementary run, thus yielding ~0.5 million solutions for each of the possible interfaces (Figure S4, *step 4*). To eliminate the most impossible solutions, the obtained ensembles were filtered by the “non-specific” post-scoring procedure requiring the following: (1) PSCA buries a significant part of its molecular surface into the nAChR interface. (2) The interaction interface exhibits complementarity of hydrophobic/hydrophilic properties. Additional requirements were a sufficient amount of intermolecular (3) hydrogen bonds and (4) salt bridges (see Section 2 and Figure S4, *step 5* for details). Detailed filtering criteria were established based on the distribution analysis (Figure S5) and are presented in Section 2. At this stage, we selected ~5000 solutions for each α4(+)/β2(−) and β2(+)/β2(−) interface and just ~1000 solutions for the β2(+)/α4(−) interface, suggesting that the latter is less preferable for ws-PSCA interaction (Figure S5).

The second “specific” post-scoring stage (Figure S4, *step 6*) was based on per-residue interaction frequency analysis. The frequency of intermolecular contacts was visualized as heatmaps (Figure S6) illuminating presumably important interactions including hydrogen bonds, ionic bonds, and stacking interactions. For both α4(+)/β2(−) and β2(+)/β2(−)-interfaces, the most frequent contacts were salt bridges between the R32 and/or R34 residues from loop II of PSCA and the D170 and/or D171 residues from the β2(−) subunits (Figure S6; the full list of frequent interactions is given in Section 2). These interactions were used as filtering criteria resulting in 19 and 14 solutions for the α4(+)/β2(−) and β2(+)/β2(−) interfaces, respectively. The best solutions for the α4(+)/β2(−) and β2(+)/β2(−) interfaces in the complex with ws-PSCA (only one in each case) were selected by visual inspection (Figure S6a,b, *right panels*).

For the β2(+)/α4(−) interface, no specific interactions with the α4(−) subunit were observed (Figure S6c), which is in accordance with generally worse interaction parameters at this interface (Figure S5). These findings suggest that the PSCA binding at this interface should be energetically disfavored and probably does not occur. Consequently, the β2(+)/α4(−) interface was excluded from further analysis.

### 3.7. MD of nAChR/ws-PSCA Complex

**Figure 6:**
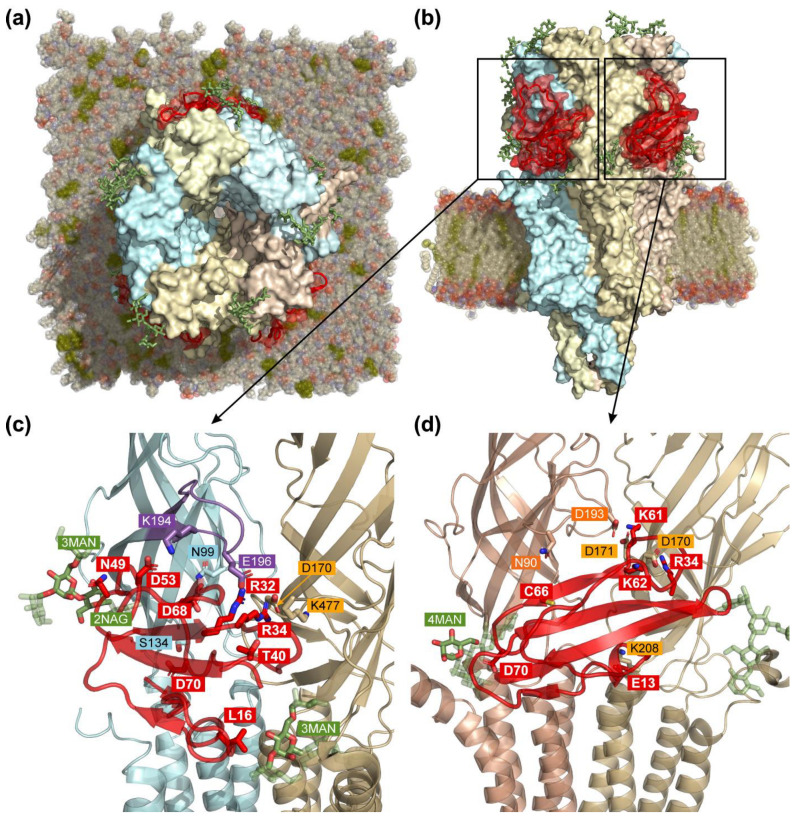
Predicted structure of the HS α4β2-nAChR/PSCA complex with three bound ws-PSCA molecules. (**a**,**b**) Top and side views on a snapshot from the 500 ns MD (replica #1) of the complex in the DOPE/DOPC/PSM/cholesterol mixed membrane. (**c**) Ws-PSCA binding to the α4(+)/β2(−) interfaces (there are two of them in the model). Ws-PSCA contacts both receptor subunits and forms hydrogen bonds with glycans (contacts are listed in Table 2). (**d**) The binding mode of ws-PSCA with the β2(+)/β2(−) interface throughout most of the MD trajectory (replica #1). β2 subunits, α4 subunits, loop C of the α4 subunit, glycans, ws-PSCA, lipids hydrophobic tails, lipids polar heads, and cholesterol are shown in tan/beige, light blue, lilac, light green, red, gray, red oxygens/blue nitrogens, and olive, respectively.

According to the results of docking simulations, both α4(+)/β2(−) and β2(+)/β2(−) interfaces can bind ws-PSCA. As HS (α4)_2_(β2)_3_-nAChR isoform contains both of these interfaces simultaneously (Figure 5c), we used the receptor in the HS stoichiometry for further MD study. The model of the HS α4β2-nAChR/ws-PSCA complex was assembled from the individual subunit pairs (interfaces) from the best docking solutions (Figure S6a,b, *right panels*). Thus, our model contains three PSCA molecules: two identically bound at the α4(+)/β2(−) interfaces and one at the β2(+)/β2(−) interface (Figure 6).

To analyze the stability of the HS α4β2-nAChR/ws-PSCA complex, we performed two 500 ns replicas of MD simulation in a lipid membrane. For most of the MD time in the first replica, PSCA was positioned parallel to the binding site surface relative to the β-sheet orientation, maintaining the key intermolecular salt bridges (see Table 2 and Table S4). The protein at the β2(+)/β2(−) interface exhibited several ionic interactions with lifetime > 80% of the total MD length, while at the α4(+)/β2(−) interface, numerous short-lived interactions were observed. The following common pattern of the receptor/PSCA interaction was revealed: the negatively charged D170 and D171 receptor residues on the β2(−) subunit, D193 on the β2(+) subunit, or E196 on the α4(+) subunit form ionic and hydrogen bonds with positively charged PSCA residues K61 (β2(+)/β2(−) interface), R34 (both interfaces), and R32 (α4(+)/β2(−) interface). For both interfaces, the interactions primarily involved the residues from loops II and III of PSCA (see Table 2 and Table S4). Additionally, long-lived hydrogen bonds (lifetime > 20%) between the ligand residues and oligosaccharides were observed across both interfaces: one at the β2(+)/β2(−) interface and three at the α4(+)/β2(−) interface (Table 2).

As indicated by the RMSD analysis (Figure S14), two ws-PSCA molecules remained stably bound to the α4(+)/β2(−) interfaces during the whole 500 ns of the first MD replica (Figure 6c). However, the ws-PSCA molecule at the β2(+)/β2(−) interface (Figure 6d) exhibited a substantial rearrangement after 430 ns (Figure S14, *red arrow*) turning approximately orthogonally to the initial binding mode (Figure S14, *inset*). In this “orthogonal” orientation, PSCA lost some of its initial contacts with the receptor retaining only ionic interactions by K61 and Y58/V181(−) and G60/D170(−) hydrogen bonds. Other interactions were lost despite the initial stability (see Table 2 and Table S4, “β2(+)/β2(−) 450–500 ns” column).

To assess the reproducibility of these findings, a second independent 500 ns MD simulation was performed. The RMSD analysis revealed a divergent behavior between the interfaces (Figure S15). Contrarily to replica #1, PSCA remained stably bound at the β2(+)/β2(−) interface, while it underwent an “orthogonal” rearrangement at the α4(+)/β2(−) interface (Figure S15, *insets*). Although the limited timescale of both 500 ns simulations precludes definitive conclusions about the global stability of the complex or the thermodynamic preference for a specific binding interface, rearrangements in both independent replicas suggest that the initial docked configurations may be non-optimal and require further optimization in mutagenesis studies.

To analyze ws-PSCA dynamics in MD, its Root Mean Square Fluctuation (RMSF) values were calculated in free and bound states (Figure S16). RMSF distribution for free ws-PSCA resembled the order parameter S^2^ calculated from ^15^N relaxation data (Figure S11). The loop regions were the most flexible, especially the loop III fragment (residues 53–60). When bound to the α4(+)/β2(−) interfaces (MD replica #1), the RMSF values for some residues significantly exceeded those of the free ligand, while no such increase was observed for the β2(+)/β2(−) interface (Figure S16). This suggests that the receptor residues interacting with the ligand at the α4(+)/β2(−) interfaces may induce additional fluctuations in bound ws-PSCA.

## 4. Discussion

The main goal of the present study was to investigate the molecular mechanisms of the PSCA action and its role in the brain function. Studies of the Ly6/uPAR proteins remain a challenging task due to their involvement in many essential processes. A significant obstacle of the Ly6/uPAR protein studies is the membrane tethering of these proteins to the cell membrane via the GPI-anchor, which significantly complicates recombinant production. Despite the fact that there are only 36 annotated genes encoding human Ly6/uPAR proteins, this protein family remains poorly studied. In this work, we produced and studied the isolated correctly folded water-soluble Ly6 domain of PSCA using a bacterial expression system (Figure 2). The validity of this approach is supported by the in vivo existence of soluble form of PSCA found in the human cerebral cortex in AD [35] and in the cerebellum of mice modeling the early stage of AD [36]. Moreover, PSCA expressed in the cerebrospinal fluid (Figure 1a) also should be soluble. Successful development of the recombinant expression system opened new possibilities for structural–functional studies of this human protein.

Previously, PSCA was shown to form a stable complex with the α4 nAChR subunit from the human cerebral cortex [35]. Here, we investigated ws-PSCA action at different nAChR subtypes containing the α4 subunit and found that ws-PSCA inhibits α4β2- and α3β2-nAChRs but has no effect on α4β4-nAChRs. This means that the β2 subunit is the crucial determinant of the PSCA/nAChR interaction. Similarity of IC_50_ values at HS and LS α4β2-nAChRs (~27 and 15 μM, respectively, Table 1, Figure 5b) suggests that the PSCA/nAChR interaction does not depend on the receptor stoichiometry.

Three-finger neurotoxins from snake venoms are structural homologs of the endogenous proteins from the Ly6/uPAR family. Many of these toxins also target nAChRs [75]. However, the toxins typically demonstrate high (nanomolar) affinity and competitively inhibit these receptors by the binding at the orthosteric site. At the same time, the human Ly6/uPAR proteins, such as Lynx1, Lypd6, SLURP-1, and SLURP-2 are nAChR modulators with low (micromolar) affinities, and do not completely inhibit the receptors acting outside of the ACh binding pocket [52,60,65,76]. From a pharmacological view, ws-PSCA is a typical modulator; it acts on nAChRs with IC_50_ of ~15–50 μM and inhibits ion currents by no more than ~70% of the control values (Figure 5).

Previous reports on the increased expression of *LY6/UPAR* genes in some mental disorders [23] encouraged us to perform a similar bioinformatic analysis for the *PSCA* expression in the brain of healthy individuals and patients with various neurological disorders using the GTEX and GEO databases. *PSCA* expression was found in all brain regions covered by the GTEX database (Figure 1a), and the *PSCA* level in the ACC, which is responsible for emotions and cognitive function [77], was upregulated in the individuals aged > 50 years (Figure 1b). Since age-related cognitive and emotional disorders are often accompanied by dysfunction of the cholinergic system in the brain [78] and loss of the ACC gray matter [79], we hypothesize the relationship between age-related increase in the *PSCA* expression, dysfunction of nAChRs, and ACC degeneration.

In AD, the increased *PSCA* level in the cerebellum and entorhinal cortex (Figure 1c) may dysregulate the cholinergic system, which controls generation of hippocampal theta oscillations, important for motor performance [80]. Thus, PSCA may be involved in impairment of coordination and navigation since both the cerebellum and entorhinal cortex provide circuits to control spatial position of the body [81]. Differential regulation of *PSCA* in MS (upregulation in the motor cortex accompanied by downregulation in the frontal and parietal cortex; Figure 1c) may be linked with PSCA pro-inflammatory function. Chronic inflammation in turn drives MS progression in the cortex: neuronal demyelination and microglia activation leads to severe neurodegeneration [82]. Upregulation of *PSCA* in the prefrontal cortex of patients with Huntington’s disease (Figure 1c) may be linked with decreased connectivity of the prefrontal cortex [83]. Notably, the cholinergic system maintains prefrontal cortex connectivity by regulation of synaptic plasticity [84], so PSCA may mediate the cholinergic dysfunction, which in turn leads to impairment of associative recognition during the disease onset. In Down syndrome, which is characterized by intellectual disability and impairments of attention, *PSCA* is upregulated in the ventral part of the medial prefrontal cortex (Figure 1c), which controls these functions in the brain [85]. In bipolar disorder we found upregulation of *PSCA* in the cerebellum (Figure 1c). The cerebellum may mediate behavioral and social deficits [86], and nAChRs are implicated in pathogenesis of autistic spectrum disorders, which share some symptoms with bipolar disorders [87]. Increase in *PSCA* expression in patients with HAD (Figure 1c) may be connected with white matter and oligodendrocyte loss [88]. Downregulation of *PSCA* in people with cocaine addictions (Figure 1c) may be connected with changes in the glutamatergic transmission. The upregulation of GluR2, GluR5, and KA2 glutamate receptors was found in patients with cocaine overdose [89], so we may assume that decreased *PSCA* level promotes nAChR-mediated activation of the glutamatergic transmission in these patients.

Dysfunction of the nicotinic receptors was described during normal aging [90] and in many mental disorders: α4β2- and α7-nAChRs are downregulated in AD [15], schizophrenia [91], and in autistic spectrum disorders [92,93], whereas upregulation of α7-nAChRs was shown in bipolar disorder [94], and upregulation or downregulation of α4β2-nACRs was revealed in obesity [95] or PD [15], respectively. Altered signaling through α7-nAChR is related to pathogenesis of MS [96], Huntington’s disease [97], HIV-associated dementia [98], eating disorders [99], and frontotemporal dementia [100]. The dysfunction of the α4β2-nAChR signaling is implicated in the development of smoking addiction [101] and obsessive–compulsive disorder [102], whereas signaling through both receptors is declined in depression [103], schizophrenia [15,104], and alcoholism [105]. Heteromeric nAChRs containing the β2 subunit are implicated in Down syndrome [106], alcohol addiction [107], and dopamine release upon cocaine consumption [108]. Mutations in the α4 and β2 subunits are characteristic for epilepsy [15]. Moreover, the interplay between α7-nAChRs and α4β2-nAChRs in the regulation of different cognitive processes is described [109]. Taking in mind α4β2-nAChR targeting by PSCA (Figure 5), these data additionally support our hypothesis about the relationship between the nAChR dysfunction during aging and in various brain pathologies with the altered *PSCA* expression identified here (Figure 1b,c). This observation may have pathophysiological significance, as successful treatment of neurological diseases depends on identification and modulation of key dysregulated molecular pathways [110,111,112]. In this context, the interaction between PSCA and nAChRs represents a novel and compelling pathway.

Neurological diseases, for which we observed altered *PSCA* expression (Figure 1c), are accompanied by neuroinflammation [68,69,70,71,72,113]. Neuroinflammation is mediated by the secretion of various pro-inflammatory factors by microglia and astrocytes [114]. The neurons, in turn, may respond to these inflammatory signals by a release of their own mediators, creating a feedback loop [115,116]. Here, we found that ws-PSCA regulates the secretion of pro- and anti-inflammatory factors implicated in the development of different neurodegenerative diseases (Figure 3). For example, upregulation by ws-PSCA pro-inflammatory adhesion molecule VCAM-1 may mediate disruption of the brain-blood barrier, serve as an AD marker [117], and may be implicated in MS pathology [118,119]. Pro-inflammatory adhesion factor E-selectin downregulated in the neurons but upregulated in the astrocytes upon incubation with ws-PSCA (Figure 3) is elevated in the cerebrospinal fluid of AD patients [120]. In MS patients, the E-selectin level is elevated only in primary but not relapsing disease [121]. Moreover, E-selectin and VCAM-1 are considered biomarkers of stroke burden [122]. EpCAM, which can mediate regulation of leukocyte adhesion upon inflammatory conditions in the brain [123], was downregulated by ws-PSCA in the neurons (Figure 3). The most pronounced effect of ws-PSCA is the dramatic increase in TNFβ secretion both by the neurons and astrocytes (Figure 3). The increased level of TNFβ was reported in MS [124] and Huntington’s disease [125], and drives neurodegeneration in the meninges [126]. TNF-β signaling is crucial for the development of ectopic lymphoid formation in the meninges. These structures attract lymphocytes and provide sustained chronic inflammation and cortical demyelination [126]. In AD, the role of TNF-β is less direct. It may act through TNFR signaling, enhancing the activation of microglia and astrocytes, potentiating the release of cytotoxic cytokines, and complement proteins [127]. Thus, based on the data obtained, PSCA can be considered pro-inflammatory regulator involved in the development of neurodegeneration. Despite this, its influence on disease-specific neuroinflammatory mechanisms remains to be elucidated.

The analysis of the different effects of ws-PSCA on neurons and astrocytes provide some insights into its pro-inflammatory effects. VCAM-1 upregulation in astrocytes causes recruitment of immune cells [128], while VCAM-1 upregulation in the neurons of PD patients is accompanied by mitochondrial dysfunction and synapse degeneration [129]. Thus, selective upregulation of VCAM-1 in neurons (Figure 3) means that PSCA may mediate neurodegeneration without the activation of the defense mechanisms mediated by astrocytes and immune cells. EpCAM regulates the cytoskeleton assembly and cell–cell interaction of epithelial cells [130], so its selective downregulation in neurons upon incubation with ws-PSCA may decrease neuronal but not astrocytic interactions. Downregulation of the astrocytic IL-12, which mediates microglial activation [131], indicates that the pro-inflammatory effect of ws-PSCA is probably not related to microglial activation. Thus, ws-PSCA may act as the pro-inflammatory factor promoting neurodegeneration and loss of neuronal contacts.

We can compare the action of ws-PSCA on neurons and astrocytes with the action of another water-soluble Ly6/uPAR modulator, –ws-Lynx1. Contrarily to PSCA, Lynx1 is downregulated in AD [132], and incubation with ws-Lynx1 stimulates astrocytes to secrete the dendritic growth factor ALCAM-1 and abolishes the secretion of pro-inflammatory factors ICAM-1, PSGL-1, VCAM-1, CD44, and NCAM-1 [37]. Thus, various endogenous Ly6/uPAR proteins can demonstrate opposite effects in neurons, astrocytes, and the inflammatory environment, suggesting their different role in the brain.

The 3D structure of ws-PSCA revealed a classical three-finger scaffold dominated by a β structure (Figure 4) consistent with the ws-PSCA classification as the member of the Ly6/uPAR family. High-amplitude mobility in the ps-ns time scale in loops II (Ala35–Leu36) and III (Cys50–Lys61) supported by low NMR structure convergence reduced order parameters (S^2^), and high RMSF values in the MD trajectory (Figure 4a,c and Figure S15) suggested participation of these fragments in the interaction with target receptors. Indeed, computer modeling (Figure 6) confirmed that the PSCA residues of loops II and III are major epitopes of the interaction with α4β2-nAChRs (Table 2). Notably, the loops of Ly6/uPAR proteins are their most variable regions responsible for functional diversity [18]. It was proposed that the high conformational plasticity of the loop regions ensures the interaction of human Ly6/uPAR proteins with multiple targets by conformational selection [133]. In this case, the observed wide distribution of the μs-ms conformational exchange processes in the ws-PSCA molecule (Figure 4c, *right*) may also be important for the functional adaptability of the protein.

Ensemble docking and MD simulations revealed electrostatic interactions between positively charged residues of ws-PSCA and negatively charged groups of α4β2-nAChR as the main factor of the complex stability (Table 2). There are two clusters of positively charged groups on the two sides of the ws-PSCA molecule (Figure 4d). The first is formed by the residues located near the tips of loops II and III (R32, R34, and K61), while the second one located in the middle of the PSCA β-structural core is formed by the residues of all three loops (K7, K44, and K62). Only the first cluster forms persistent intermolecular ionic bridges upon the ws-PSCA binding to both of the following possible interfaces: α4(+)/β2(−) and β2(+)/β2(−) (Figure 6, Table 2). In the complementary β2(−) subunit, these electrostatic contacts involve the residues D170 and D171, whereas in the primary α4(+) or β2(+) subunits, the contacts are formed with the receptor’s residues E196 or D193, respectively. Only the α4(+)/β2(−) interface contains the orthosteric agonist binding site located under the C-loop (T192–D204; Figure 6c, *lilac*) of the primary α4(+) subunit. Ws-PSCA binds to the nAChR surface directly below the C-loop and interacts peripherally with it by the R32 and D53 residues from loops II and III, respectively, to form the ionic bridges with the C-loop residues K194 and E196 (Figure 6c). In this interaction, the ws-PSCA molecule does not penetrate the orthosteric site and could allosterically modulate the agonist binding and receptor activation/inactivation. This mode of the PSCA/nAChR interaction resembles the interaction modes proposed for other Ly6/uPAR modulators (Lynx1, Lypd6, SLURP-1, and SLURP-2) and snake toxin WTX, which also depend on charge-driven recognition of the receptor’s (+)/(−)-subunit interfaces by the structurally flexible loops of the three-finger molecules [52,60,65,76,134]. On the other hand, the ws-PSCA binding to the β2(+)/β2(−) interface may induce some changes in the packing of the receptor’s subunits and allosterically affect the agonist binding and/or receptor activation/inactivation.

It is important to note the inherent limitations of our study. Inhibitory activity of ws-PSCA on α3β2-nAChRs (Figure 5b) revealed a new, previously not considered target of this neuromodulator. This raises the following question: which receptor is the primary target of PSCA in the brain? As mentioned above, α4β2-nAChR is one of the most abundant subtypes of the nicotinic receptors in the brain [16], while α3β2-nAChRs are expressed mainly in the cortex, striatum, and cerebellum [135]. It is likely that PSCA can regulate the cholinergic signaling in different regions of the brain by the interaction with both α4β2- and α3β2-nAChRs, although the existence of other inflammation-related PSCA targets cannot be excluded and requires further study.

We have previously suggested that the transition from the GPI-anchored to soluble form of PSCA upon Aβ accumulation in the brain is related to AD progression [36]. The inhibitory effect of ws-PSCA on α4β2-nAChRs observed here may reflect the activity of the soluble protein in the AD brain. Perhaps α4β2-nAChRs downregulated by Aβ [135] experience additional downregulation by an increased amount of soluble PSCA in the brain. However, the function of membrane-tethered PSCA can be different from that of soluble PSCA, at least due to space-specific expression. The reason and role of PSCA and ws-PSCA increase in AD and other pathologies remain unknown and should be further investigated.

Another limitation of this study is that we did not determine the sex of the newborn rats to obtain primary cultures of the neurons and astrocytes. Thus, we cannot assess the sex-specific effects of ws-PSCA. Notably, estrogen and testosterone act differently on the astrocyte differentiation and morphology [136]. In our case, hormonal effects are not evident in vitro, but the sex-specific effects of PSCA are likely possible in vivo and require further attention.

The pharmacological profile of ws-PSCA was characterized by means of the common electrophysiology approach using *Xenopus laevis* oocytes expressing specific nAChR subtypes. A recognized limitation of this heterologous expression system is the absence of native neuronal membrane environment, including potential intramolecular interactions with endogenous molecular partners and auxiliary proteins that may modulate nAChR function in the brain.

Recently, artificial intelligence-based technologies have been proposed to revolutionize structural biology and facilitate pharmacological studies. To assess the applicability of this approach to the study of the Ly6/uPAR modulators, we compared the determined structure of ws-PSCA and the modeled α4β2-nAChR/ws-PSCA complex with AlphaFold3 server (https://alphafoldserver.com) predictions [137]. The comparison revealed quite good correspondence in the overall ws-PSCA structure and position of the individual elements of β-structure (Figure S17). However, large discrepancies were found in the conformations of loops I and III. AlphaFold3 over-stabilizes these dynamically mobile regions and suggests the presence of two additional β-strands in these loops, which were not observed in the experiment. One of the critical limitations of the AlphaFold3 model is its prediction of static protein structures, which fails to capture a dynamic behavior of flexible protein regions and disordered loops [137]. Ly6/uPAR proteins like Lynx1, Lypd6, SLURP-1, SLURP-2, and PSCA possess flexible loop regions, whose dynamics could be the important determinants of ligand–receptor interactions [133]. Therefore, the AlphaFold3 model should be carefully evaluated for the prediction of the Ly6/uPAR protein structures. When modeling the α4β2-nAChR/ws-PSCA complex, AlphaFold3 placed three ws-PSCA molecules on three β2 subunits presented in the HS form of the receptor (Figure S18), which agrees with the data on the predominant role of the β2 subunit in the interaction. At the same time, the proposed solutions turned out to be unrealistic: ws-PSCA molecules bind to the membrane interface of the receptor, and the interaction interface in each case includes only the β2 subunit, but not the whole binding site, which should involve the following two subunits: primary (+) and complimentary (–).

The MD runs performed here were relatively short (500 ns each) and do not allow reliable prediction of the stability or instability of the PSCA/nAChR complex. Nevertheless, even this time scale of the simulations indicates the possibility of rearrangement of the complex and reorientation of PSCA “perpendicular” to the receptor surface, in contrast to the results obtained with ensemble docking. Ultimately, a reliable test of the obtained models, and especially the proposed binding interfaces, requires further experimental confirmation through structural and/or mutagenetic studies.

## 5. Conclusions

Here, we combined bioinformatic, protein engineering, cell biology, biophysical, and functional studies to elucidate the PSCA structure, pharmacology, and possible role in modulation of the cholinergic signaling in the brain and trace the possible association of PSCA expression with CNS diseases. This integrative approach bridges together clinical manifestations of aging and neurodegenerative disorders with the targeting of β2-subunit-containing nAChRs by PSCA. The study provides a new look on the progression of mental disorders associated with the dysfunction of neuronal nAChRs. Perhaps the development of these diseases is related not with altered nAChR expression, but with disbalanced expression of the nAChR modulators from the Ly6/uPAR family (such as Lynx1, Lypd6, or PSCA), which could, in turn, affect nAChR function and lead to dysregulation of the brain cholinergic system. Another important finding of this study is that PSCA acts as a pro-inflammatory factor, which could mediate neuroinflammation, neurodegeneration, and loss of neuronal contacts. Modern therapeutic strategies for neurodegenerative diseases focus on the modulation of specific pathological pathways, including neuroinflammation. Thus, the targeting of the PSCA/nAChR interaction can be a promising future therapeutic strategy. Elimination of PSCA excess or PSCA mimetics may be used to control the cholinergic system in some pathologies associated with downregulation or upregulation of α4β2-nAChRs, respectively.

## Figures and Tables

**Table 1:** Ws-PSCA inhibition curve parameters for HS/LS α4β2- and α3β2-nAChRs.

Receptor Type	IC_50_, μM	Maximal Inhibition, %	nH
HS α4β2	27 ± 13	31 ± 7 ^1^	1.4 ± 0.6
LS α4β2	15 ± 13		0.7 ± 0.2
α3β2	50 ± 25		1.2 ± 0.4

^1^ All three datasets were fitted simultaneously using a single level of maximal inhibition.

**Table 2:** Key α4β2-nAChR and ws-PSCA residues participating in the binding at the α4(+)/β2(−) and β2(+)/β2(−) interfaces from the MD simulation: replica #1.

ws-PSCA Residue	High-Sensitive α4β2-nAChR, Primary (+)/Complementary (−) Subunits, MD Time ^1^					
	α4(+)/β2(−), 30–500 ns		β2(+)/β2(−), 30–400 ns		β2(+)/β2(−), 450–500 ns	
	(+)	(−)	(+)	(−)	(+)	(−)
**ws-PSCA Loop I**						
E13				**K208 (I, H)**		
L16		MAN (H; N460)				
**ws-PSCA Loop II**						
R32	E196 (I, H)	D170 (I, H)				
R34	**E196 (I, H)**	**D170 (I, H)**		**D170 (I, H)**		
T40		K477 (H)				
**ws-PSCA Loop III**						
N49	**MAN (H; N146)** NAG (H; N146)					
D53	K194 (I, H)					
Y58						V181 (H)
G60						**D170 (H)**
K61			**D193 (I, H)**	**D171 (I, H)**	**D193 (H, I)**	D170 (H, I) **D171 (H, I)**
K62			D193 (I, H)			
N63	K194 (H)					
C66			**N190 (I, H)**			
D68	**N99 (H)** S132 (H)					
D70	**S134 (H)**		MAN (H; N143)			

^1^ Receptor residues are annotated by the interaction type in parentheses: H—hydrogen bonds, I—ionic bridges. Primary (+) and complementary (−) subunits are described in separate columns; the latter are over gray background. Monosaccharide abbreviations: MAN—mannose, NAG—N-acetylglucosamine. For monosaccharides, the parent glycosylated residue number is indicated in parentheses. The data for the β2(+)/β2(−) interface are presented for two trajectory segments: initial (30–400 ns, before the ligand rearrangement) and final (450–500 ns, post-rearrangement). The table includes all contacts persisting for ≥20% of the simulation time (470 ns for α4(+)/β2(−), 370 ns and 50 ns for β2(+)/β2(−) interfaces). nAChR residues maintaining interactions for ≥50% of the simulation time are highlighted in bold. The full interaction data is presented in Table S4.

## Data Availability

The data presented in this study are available in Supporting Information. MD simulation (setup files and trajectory) of ws-PSCA in the modeled complex with HS (α4)_2_(β2)_3_-nAChR is available at the Zenodo repository: https://doi.org/10.5281/zenodo.17038800.

## Supplementary Materials

The following supporting information can be downloaded at: https://www.mdpi.com/article/10.3390/biom15101381/s1, Table S1: PSCA expression in the brain of patients with neurological and mental disorders; Table S2: Comparison of PSCA levels in different regions of the brain according to Kruskal–Wallis test followed by Dunn’s post hoc test; Table S3: Statistics for the best CYANA structures of ws-PSCA; Table S4: Pairwise interaction data from MD simulation of the a4b2-nAChR/PSCA complex, describing all observed hydrogen bonds, ionic interactions, π-cation contacts, and stacking interactions; Figure S1: PSCA expression in the brain of patients with neurological and neuropsychiatric disorders; Figure S2. The regression curves used for interpolation of adhesion molecules concentration; Figure S3. The gating strategy for the Legendplex adhesion molecules immunoassay; Figure S4: Computational modeling of the HS α4β2-nAChR/ws-PSCA complex through ensemble docking and post-scoring; Figure S5: Post-scoring metrics for ws-PSCA docking ensembles into three HS/LS α4β2-nAChR dimeric interfaces; Figure S6: Specific post-scoring of α4β2-nAChR/ws-PSCA ensemble docking based on intermolecular contacts frequency analysis; Figure S7: Uncropped and unedited original image of the SDS-PAGE gel of the purified ws-PSCA (MW ~ 8.3 kDa); Figure S8: ^15^N-HSQC NMR spectrum of ^13^C,^15^N-labeled ws-PSCA; Figure S9: NMR data define the secondary structure of ws-PSCA in aqueous solution; Figure S10: Scheme of the contacts between β-strands observed in the NOESY spectra; Figure S11: ^15^N relaxation data and results of the ‘model-free’ analysis of ws-PSCA; Figure S12: Normalized intensities of signals of ws-PSCA in the 3D HNCO spectrum plotted versus ws-PSCA sequence; Figure S13: Average currents evoked by 5 s pulses of ACh on α3β2, α4β4, and α4β2 LS/HS nAChRs in the absence or presence of 100 μM ws-PSCA; Figure S14: Backbone RMSD of ws-PSCA in the complex with HS (α4)_2_(β2)_3_-nAChR, derived from MD replica #1; Figure S15: Backbone RMSD of ws-PSCA in the complex with HS (α4)2(β2)3-nAChR, derived from MD replica #2; Figure S16: RMSF for free and nAChR-bound ws-PSCA under different conditions; Figure S17: Comparison of the ws-PSCA structures obtained by NMR and predicted from the sequence by AlphaFold3; Figure S18: Comparison of the models of HS (α4)_2_(β2)_3_-nAChR in the complex with three ws-PSCA molecules obtained by ensemble docking/MD simulations and constructed using AlphaFold3.
