# Supplementary material for: Pro-Inflammatory Protein PSCA Is Upregulated in Neurological Diseases and Targets β2-Subunit-Containing nAChRs
Source: Biomolecules. 2025 Sep 28;15(10):1381. doi: 10.3390/biom15101381 (PMC12562601; doi:10.3390/biom15101381)
Supplement: Supplementary file 1 [file biomolecules-15-01381-s001.zip › biomolecules-3807827-supplementary.pdf]

biomolecules

Supporting Information

Pro-inflammatory Protein PSCA is Upregulated in Neurological Diseases and Targets β2-Subunit-Containing nAChRs

Mikhail A. Shulepko [1,#], Yuqi Che [1,#], Alexander S. Paramonov [2,#], Milita V. Kocharovskaya [2,3], Dmitrii S. Kulbatskii [2], Anisia A. Ivanova [3], Anton O. Chugunov [2,3,4], Maxim L. Bychkov [2], Artem V. Kirichenko [2], Zakhar O. Shenkarev [2,3], Mikhail P. Kirpichnikov [2,4], Ekaterina N. Lyukmanova [1,2,3,4,*]

1 Faculty of Biology, Shenzhen MSU-BIT University, 518172 Shenzhen, China.
2 Shemyakin-Ovchinnikov Institute of Bioorganic Chemistry, Russian Academy of Sciences, 117997 Moscow, Russia.
3 Moscow Center for Advanced Studies, 123592 Moscow, Russia.
4 Scientific Research Institute for Systems Biology and Medicine, 117246 Moscow, Russia
5 Interdisciplinary Scientific and Educational School of Moscow University «Molecular Technologies of the Living Systems and Synthetic Biology», Faculty of Biology, Lomonosov Moscow State University, 119234 Moscow, Russia.
# These authors with equal contribution
* Correspondence: lyukmanova_ekaterina@smbu.edu.cn

Table S1. Comparison of *PSCA* levels in the different brain regions according to Kruskal-Wallis test followed by Dunn's post hoc test.

Brain regions compared	Mean rank diff.	Summary*	Adjusted *p* Value
Hippocampus vs. ACC	-223.2		0.151
Hippocampus vs. Front. cortex	-179		0.7021
Hippocampus vs. Amygdala	-36.63		>0.9999
Hippocampus vs. Caudate	186.4		0.3783
Hippocampus vs. Nucleus accumbens	68.68		>0.9999
Hippocampus vs. Putamen	60.42		>0.9999
Hippocampus vs. Hypothalamus	246.9	#	0.0313
Hippocampus vs. Substantia nigra	356.3	#	0.0003
Hippocampus vs. Cerebellum	111.2		>0.9999
Hippocampus vs. Spinal cord	409.5	#	<0.0001
Hippocampus vs. Pituitary gland	221.1	#	0.0482
ACC vs. Front. cortex	44.24		>0.9999
ACC vs. Amygdala	186.6		>0.9999
ACC vs. Caudate	409.6	#	<0.0001
ACC vs. Nucleus accumbens	291.9	#	0.0018
ACC vs. Putamen	283.6	#	0.006
ACC vs. Hypothalamus	470.1	#	<0.0001
ACC vs. Substantia nigra	579.5	#	<0.0001
ACC vs. Cerebellum	334.4	#	0.0001
ACC vs. Spinal cord	632.7	#	<0.0001
ACC vs. Pituitary gland	444.3	#	<0.0001
Front. cortex vs. Amygdala	142.3		>0.9999
Front. cortex vs. Caudate	365.3	#	<0.0001
Front. cortex vs. Nucleus accumbens	247.6	#	0.0126
Front. cortex vs. Putamen	239.4	#	0.0368
Front. cortex vs. Hypothalamus	425.9	#	<0.0001
Front. cortex vs. Substantia nigra	535.2	#	<0.0001
Front. cortex vs. Cerebellum	290.1	#	0.0009
Front. cortex vs. Spinal cord	588.5	#	<0.0001
Front. cortex vs. Pituitary gland	400.1	#	<0.0001
Amygdala vs. Caudate	223		0.1444
Amygdala vs. Nucleus accumbens	105.3		>0.9999
Amygdala vs. Putamen	97.05		>0.9999
Amygdala vs. Hypothalamus	283.5	#	0.012
Amygdala vs. Substantia nigra	392.9	#	0.0001
Amygdala vs. Cerebellum	147.8		>0.9999
Amygdala vs. Spinal cord	446.2	#	<0.0001
Amygdala vs. Pituitary gland	257.7	#	0.0185
Caudate vs. Nucleus accumbens	-117.7		>0.9999
Caudate vs. Putamen	-125.9		>0.9999
Caudate vs. Hypothalamus	60.56		>0.9999
Caudate vs. Substantia nigra	169.9		>0.9999
Caudate vs. Cerebellum	-75.2		>0.9999

Caudate vs. Spinal cord	223.2		0.124
Caudate vs. Pituitary gland	34.76		>0.9999
Nucleus accumbens vs. Putamen	-8.259		>0.9999
Nucleus accumbens vs. Hypothalamus	178.2		0.5147
Nucleus accumbens vs. Substantia nigra	287.6	#	0.0081
Nucleus accumbens vs. Cerebellum	42.48		>0.9999
Nucleus accumbens vs. Spinal cord	340.9	#	0.0001
Nucleus accumbens vs. Pituitary gland	152.4		0.8704
Putamen vs. Hypothalamus	186.5		0.5061
Putamen vs. Substantia nigra	295.8	#	0.0089
Putamen vs. Cerebellum	50.74		>0.9999
Putamen vs. Spinal cord	349.1	#	0.0002
Putamen vs. Pituitary gland	160.7		0.8587
Hypothalamus vs. Substantia nigra	109.3		>0.9999
Hypothalamus vs. Cerebellum	-135.8		>0.9999
Hypothalamus vs. Spinal cord	162.6		>0.9999
Hypothalamus vs. Pituitary gland	-25.8		>0.9999
Substantia nigra vs. Cerebellum	-245.1		0.0731
Substantia nigra vs. Spinal cord	53.29		>0.9999
Substantia nigra vs. Pituitary gland	-135.1		>0.9999
Cerebellum vs. Spinal cord	298.4	#	0.0023
Cerebellum vs. Pituitary gland	110		>0.9999
Spinal cord vs. Pituitary gland	-188.4		0.465

* # ($p < 0.05$) indicates the significant difference between the data groups by Kruskal-Wallis test followed by Dunn's post hoc test.

Table S2. *PSCA* **expression in the different brain regions of patients with neurological and mental disorders.**

Brain region	LFC*	*p***	t***	B****	Patient number		GEO ID (GSE)
					healthy	diseased	
**Alzheimer disease**							
**Temporal cortex**	-0.020	0.715	-0.368	-5.881	19	10	36980
**Hippocampus**	-0.079	0.366	-0.925	-5.600	10	8	
**Entorhinal cortex (medium stage)**	-0.168	0.068	-1.856	-4.271	24	37	118553
**Temporal cortex (medium stage)**	0.0687	0.443	0.771	-5.799	31	32	
**Frontal cortex (medium stage)**	-0.120	0.187	-1.335	-5.117	23	33	
**Cerebellum (medium stage)**	0.246	0.018	2.439	-3.118	22	32	
**Entorhinal cortex (severe)**	0.219	0.026	2.282	-4.199	24	37	
**Temporal cortex (severe)**	0.084	0.292	1.061	-5.841	31	52	
**Frontal cortex (severe)**	-0.0772	0.376	0.891	-5.653	23	43	
**Cerebellum (severe)**	0.063	0.468	0.730	-5.695	22	38	
**Multiple sclerosis**							
**Frontal cortex**	-0.598	0.00001	-4.694	3.008	21	47	131282
**Motor cortex**	0.172	0.576	0.565	-6.040	10	20	135511
**Parietal cortex**	-0.536	0.002	-3.138	-1.715	21	54	131282
**Frontal cortex (lesion)**	-0.376	0.014	-2.565	-4.432	21	21	131282
**Motor cortex (lesion)**	0.768	0.004	3.053	-2.163	10	20	135511
**Parietal cortex (lesion)**	-0.536	0.002	-3.138	-1.715	21	16	131282
**Parkinsons' disease**							
**Frontal cortex (medium stage)**	-0.058	0.746	0.178	-0.324	23	18	216281
**Frontal cortex (severe)**	0.022	0.897	0.168	0.129	23	22	
**Substantia nigra**	0.316	0.543	0.620	-4.610	9	6	42966
**Huntington's disease**							
**Prefrontal cortex**	0.087	3.54 e^{-10}	6.480	12.241	157	157	33000
**Motor cortex**	0.483	0.139	0.327	1.479	7	7	79666
**Cingulate**	-0.393	0.344	0.415	-0.946	23	9	242195
**Choroid plexus**	-0.228	0.353	-0.971	-5.289	3	6	110226
**Down syndrome**							
**Ventramedial prefrontal cortex**	0.826	0.0004	4.418	0.210	7	7	59630
**Dorsolateral prefrontal cortex**	0.343	0.071	1.883	-4.3838	12	12	
**Inferior temporal cortex**	-0.234	0.595	-0.546	-5.918	5	5	
**Visual cortex**	0.008	0.964	0.714	-5.588	11	11	
**Olfactory cortex**	0.183	0.510	0.676	-5.703	6	6	
**Hippocampus**	-0.123	0.810	-0.246	-5.477	3	3	
**Cerebellum**	-0.112	0.442	-0.783	-5.865	10	10	
**Autism**							
**Dorsolateral prefrontal cortex**	-0.310	0.354	0.335	-0.926	39	13	102741
**Cerebellum**	-0.051	0.219	-1.260	-5.355	12	14	38322
**Occipital lobe**	0.068	0.054	2.187	-3.768	6	4	
**Bipolar disorder**							

**Dorsolateral prefrontal cortex**	0.087	0.687	0.217	0.403	24	24	80655
**Hippocampus**	0.005	0.941	0.075	-5.190	18	17	53987
**Cerebellum**	0.067	0.032	2.184	-3.832	50	13	35978
**Associative striatum**	0.039	0.425	0.807	-5.085	18	17	53987
**Anterior cingulate gyrus**	0.325	0.124	0.211	1.538	24	24	80655
**Nucleus accumbens**	0.379	0.132	0.252	1.508	22	22	
**HIV-associated dementia**							
**Frontal cortex**	0.242	0.105	1.730	-5.079	6	7	35864
**Frontal cortex (encephalitis)**	0.136	0.377	0.916	-6.0344	6	5	
**Basal ganglia**	-0.245	0.127	-1.621	-4.633	6	7	
**Basal ganglia (encephalitis)**	-0.191	0.248	-1.212	-5.627	6	5	
**White matter**	0.195	0.100	1.762	-4.320	6	7	
**White matter (encephalitis)**	0.805	$2.64\ e^{-05}$	6.702	2.766	6	5	
**Major depressive disorder**							
**Dorsolateral prefrontal cortex**	-0.181	0.436	0.232	-0.779	24	23	80655
**Hippocampus**	0.043	0.474	0.723	-5.020	18	17	53987
**Cerebellum**	-0.048	0.267	-1.120	-5.171	50	13	35978
**Associative striatum**	-0.011	0.831	-0.215	-5.339	18	16	53987
**Anterior cingulate gyrus**	0.204	0.369	0.227	0.898	24	24	80655
**Nucleus accumbens**	0.263	0.313	0.260	1.010	22	22	
**Schizophrenia**							
**Dorsolateral prefrontal cortex**	0.138	0.490	0.200	0.689	24	24	80655
**Hippocampus**	-0.005	0.950	-0.063	-6.087	18	15	53987
**Cerebellum**	-0.007	0.818	-0.231	-5.974	50	44	35978
**Associative striatum**	0.094	0.127	1.5560	-4.623	18	18	
**Anterior cingulate gyrus**	0.391	0.077	0.221	1.770	24	24	80655
**Nucleus accumbens**	0.286	0.254	0.2508	1.141	23	22	
**Normal ageing**							
**Frontal cortex**	0.006	0.924	0.096	-6.349	9	18	48350
**Hippocampus**	0.127	0.360	-2.161	-3.577	9	18	
**Alcoholism**							
**Frontal cortex**	-0.203	0.291	-1.117	-4.590	5	4	53808
**Motor cortex**	-0.119	0.390	-0.903	-4.630	4	4	
**Hippocampus**	-0.018	0.793	-0.264	-5.602	8	8	44456
**Smoking**							
**Nucleus accumbens**	0.047	0.747	0.146	0.323	171	50	171936
**Cocaine addiction**							
**Midbrain**	-0.066	0.030	-2.226	-3.685	30	30	54839
**Eating disorders**							
**Frontal cortex**	0.006	0.305	1.030	-6.479	102	15	60190
**Obsessive compulsive disorder**							
**Dorsolateral prefrontal cortex**	0.024	0.226	3.807	-0.197	102	16	60190
**Frontotemporal dementia**							
**Choroid plexus**	0.187	0.271	1.149	-5.326	6	4	110226

Data highlighted by red/blue means significant up-/down-regulation of *PSCA* in comparison to healthy patients

*LFC is Log2 expression change as compared to the expression level in healthy donors.

***p* – *p*-value, according to two-sided Mann-Whitney u test.

***t – size of the difference relative to the variation in data.

****B – log-odds of data transformation characterizing data distribution.

Table S3. Statistics for the best CYANA structures of ws-PSCA

**Distance and Angles Restraints**	
Total NOE contacts	524
Intraresidual	158
Interresidual:	
Sequential ($\lvert i-j \rvert = 1$)	172
Medium-range ($1 < \lvert i-j \rvert < 4$)	32
Long-range ($\lvert i-j \rvert \geq 4$)	162
Torsion angle restraints	
Angles φ	52
Angles χ_1	23
Hydrogen bonds restraints (bonds/upper/lower)	28/56/56
S-S bond restraints (bonds/upper/lower)	5/15/15
Total restraints/per residue:	728/9.6
**Statistics for calculated structures**	
Structures calculated/selected	400/20
CYANA target function (Å^2)	1.50 ± 0.21
Violations of restraints	
Upper (> 0.2 Å)	3
Upper (> 0.4 Å)	0
Lower	0
Van der Waals (>0.2 Å)	0
Dihedral angles (> 5°)	0
RMSD (stable regions Leu1-Ile33, Val41-Cys50, Asn63-Ala74), (Å)	
Backbone	0.71 ± 0.13
Heavy atoms	1.41 ± 0.17
RMSD (all atoms), (Å)	
Backbone	1.78 ± 0.43
All heavy atoms	2.66 ± 0.45

Table_S4.xlsx

Table S4. Pairwise interaction data from MD simulation of the nAChR/PSCA complex, documenting all observed hydrogen bonds, ionic interactions, π-cation contacts, and stacking interactions. The data is organized into three worksheets corresponding to different binding interfaces: one sheet for the α4(+)/β2(−) interface and two sheets for the β2(+)/β2(−) interface: before and after the ligand's rearrangement. Each row represents a single pairwise interaction:

●Resname lig / Resname rec: Residue names (ligand/receptor) or monosaccharide involved

●Ires lig / Ires rec: Residue indices (ligand/receptor) forming the interaction

●Type: Interaction type (hydrogen bond, ionic, π-cation, stacking)

●Subunit: Principal subunit (+) or complementary subunit (−)

●Glyco-residue:

○For monosaccharides: Parent glycosylated residue number

○For amino acids: "." (not applicable)

●LifeTime%: Contact occurrence (fraction of simulation frames where the interaction exists)

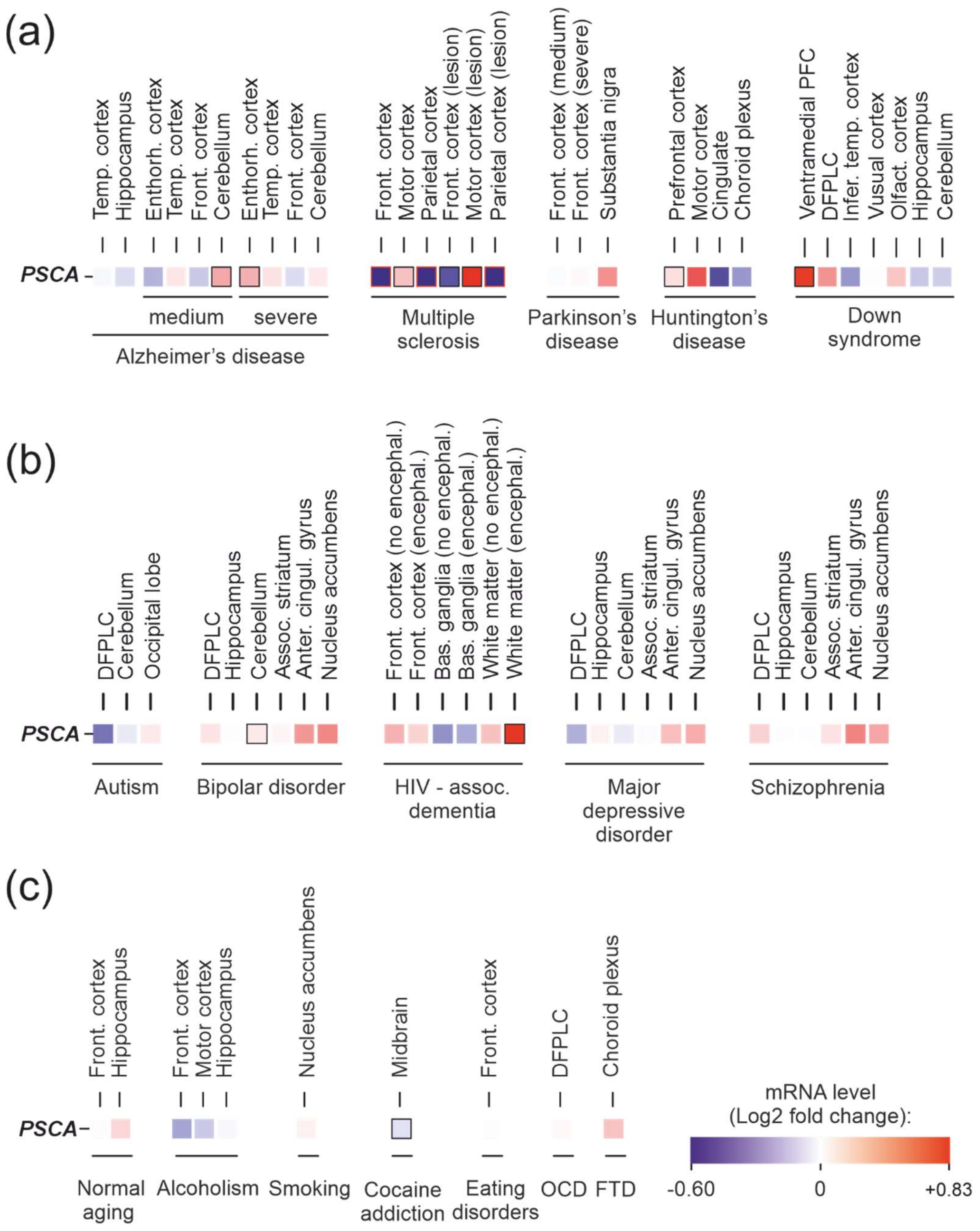

Figure S1. PSCA expression in the brain of patients with neurological and neuropsychiatric disorders. Data were downloaded from the Gene Expression Omnibus database and analyzed by Geo2R. Significant changes in comparison with donors without designated diseases by two-sided Mann-Whitney u-test are shown by black/red frames. For the dataset accession numbers, number of patients, p values, and statistical details, see Table S2.

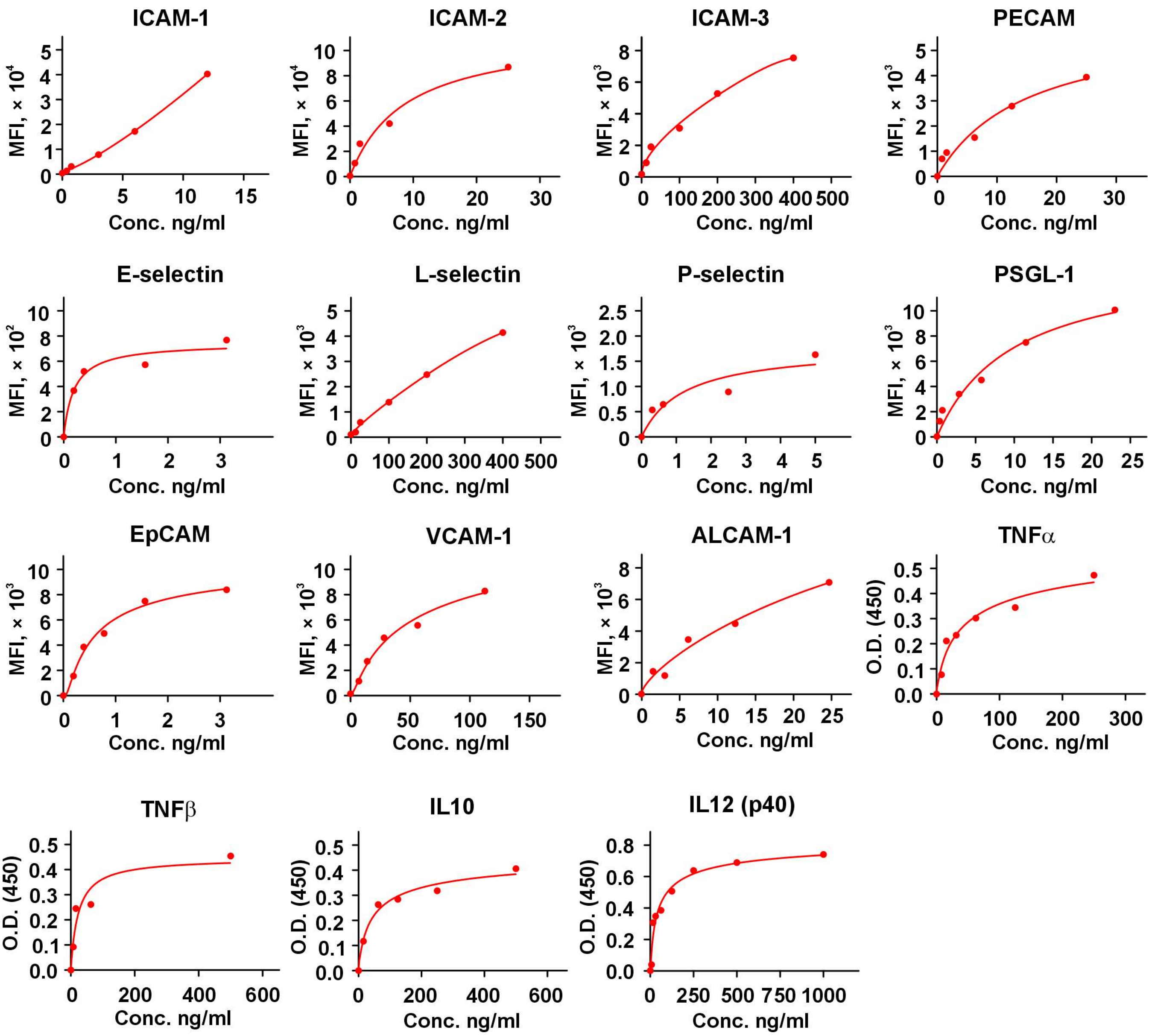

Figure S2. The regression curves used for interpolation of adhesion molecules concentration in Legendplex adhesion molecules immunoassays (see methods for details).

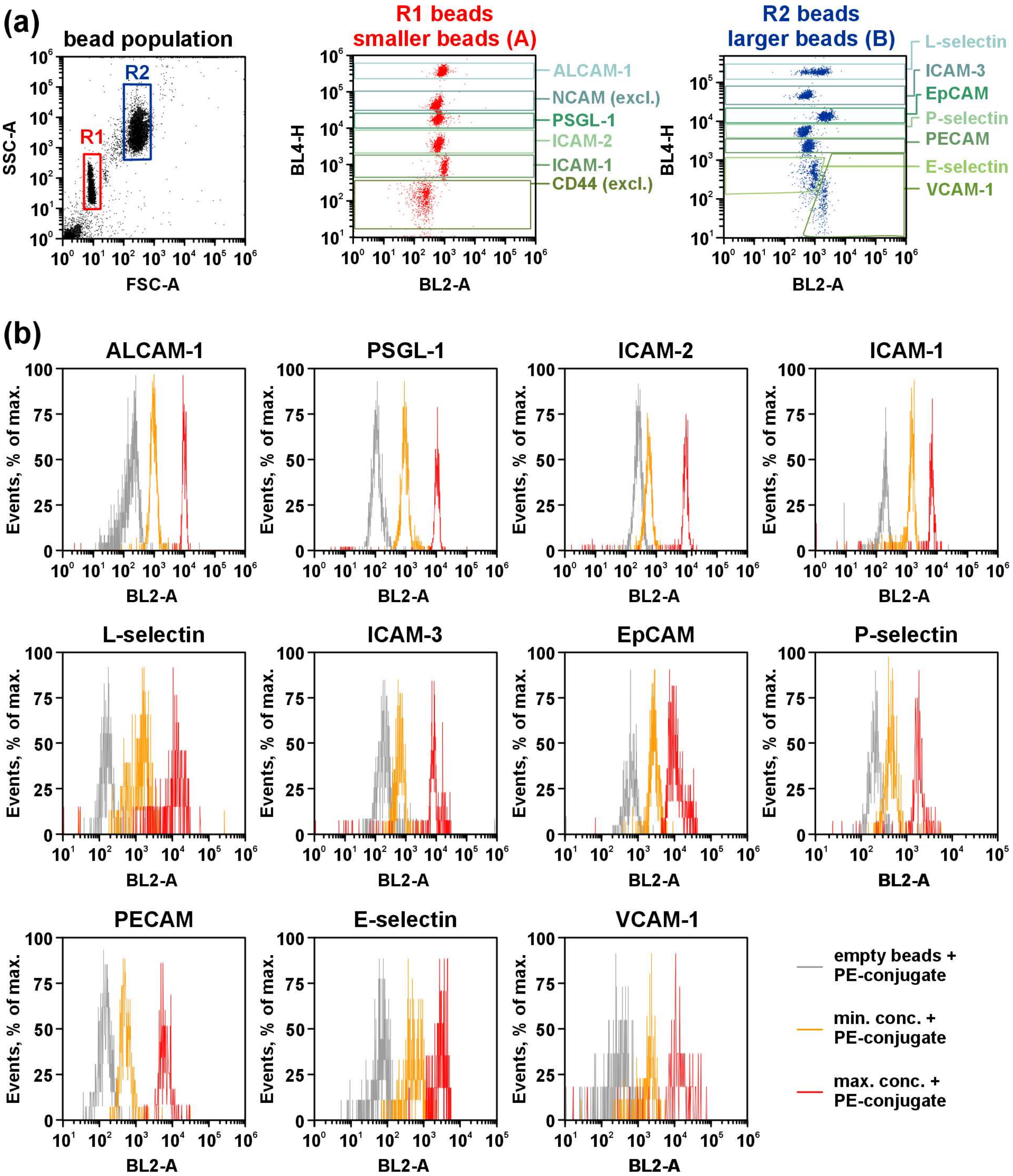

Figure S3. The gating strategy (a) and dynamic range (b) for Legendplex adhesion molecules immunoassay (Cat #740945, Biolegend). The concentrations for the curve fit are in Figure S2.

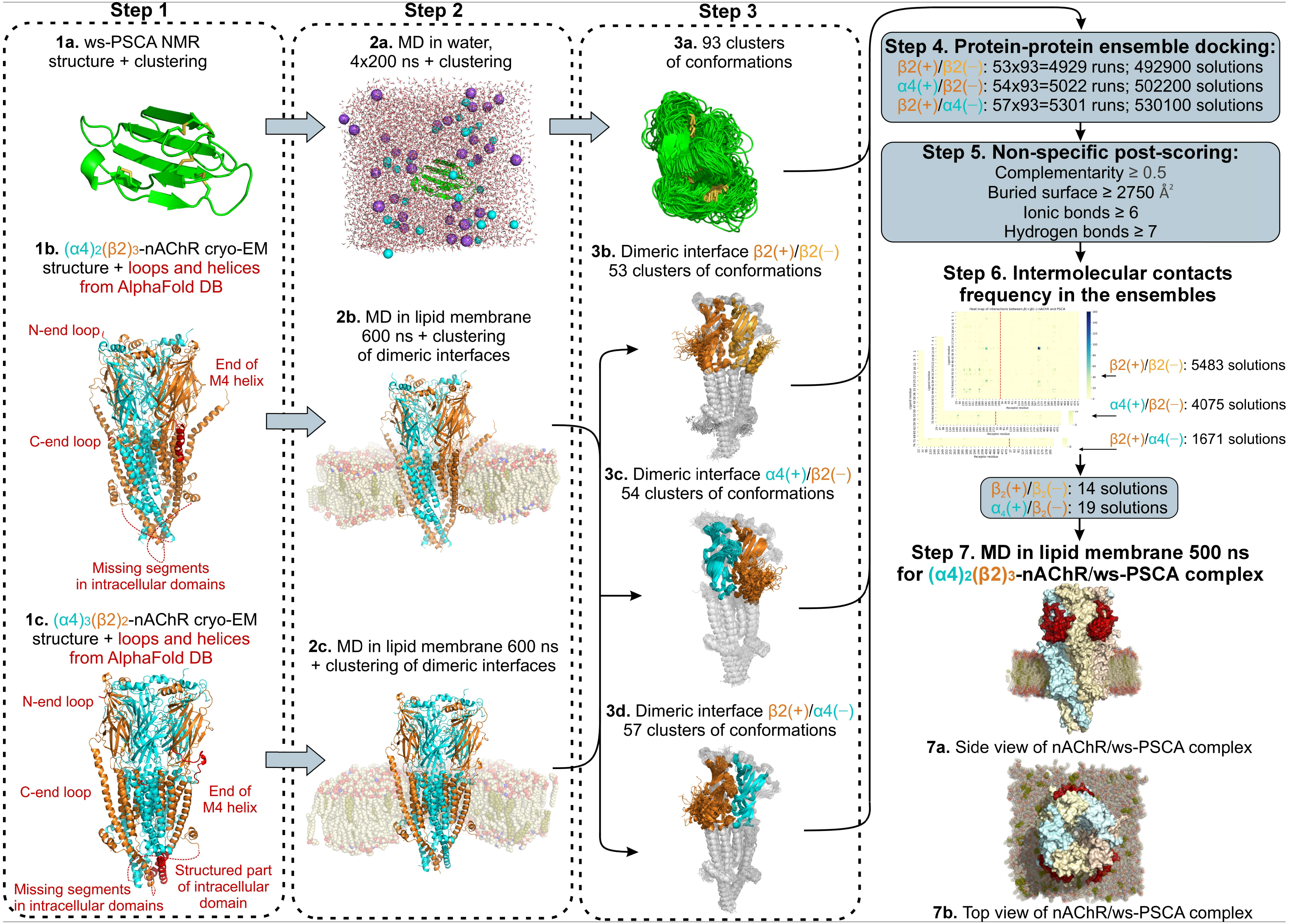
Step 1
1a. ws-PSCA NMR structure + clustering
1b. (α4)2(β2)3-nAChR cryo-EM structure + loops and helices from AlphaFold DB
N-end loop
End of M4 helix
C-end loop
Missing segments in intracellular domains
1c. (α4)3(β2)2-nAChR cryo-EM structure + loops and helices from AlphaFold DB
N-end loop
C-end loop
End of M4 helix
Structured part of intracellular domain
Missing segments in intracellular domains
Step 2
2a. MD in water, 4x200 ns + clustering
2b. MD in lipid membrane 600 ns + clustering of dimeric interfaces
2c. MD in lipid membrane 600 ns + clustering of dimeric interfaces
Step 3
3a. 93 clusters of conformations
3b. Dimeric interface β2(+)/β2(−) 53 clusters of conformations
3c. Dimeric interface α4(+)/β2(−) 54 clusters of conformations
3d. Dimeric interface β2(+)/α4(−) 57 clusters of conformations
Step 4. Protein-protein ensemble docking:
β2(+)/β2(−): 53x93=4929 runs; 492900 solutions
α4(+)/β2(−): 54x93=5022 runs; 502200 solutions
β2(+)/α4(−): 57x93=5301 runs; 530100 solutions
Step 5. Non-specific post-scoring:
Complementarity ≥ 0.5
Buried surface ≥ 2750 Å²
Ionic bonds ≥ 6
Hydrogen bonds ≥ 7
Step 6. Intermolecular contacts frequency in the ensembles
β2(+)/β2(−): 5483 solutions
α4(+)/β2(−): 4075 solutions
β2(+)/α4(−): 1671 solutions
β2(+)/β2(−): 14 solutions
α4(+)/β2(−): 19 solutions
Step 7. MD in lipid membrane 500 ns for (α4)2(β2)3-nAChR/ws-PSCA complex
7a. Side view of nAChR/ws-PSCA complex
7b. Top view of nAChR/ws-PSCA complex

Figure S4. Computational modeling of the HS α4β2-nAChR/ws-PSCA complex through ensemble docking and post-scoring. The workflow is divided into distinct phases: Steps 1–3: preparation for ensemble docking of ws-PSCA (a) and α4β2-nAChR (b–d); Step 4: ensemble docking; Steps 5–6: post-scoring; and Step 7: MD of nAChR/ws-PSCA complex. **Step 1: Experimental structures** of both ws-PSCA and HS/LS α4β2-nAChRs are taken for conformational modeling. For ws-PSCA NMR structure, determined in this work (1a), four representative conformations were selected. For HS/LS α4β2-nAChRs (1b, 1c), missing structural elements (*red*) were rebuilt according to AlphaFoldDB and MODELLER models (see text). α4-subunit are shown in *cyan*, β2-subunit — in *orange.* **Step 2: MD simulations** were conducted for ws-PSCA in aqueous solution (2a: cumulative trajectory of 800 ns; sodium ions are *purple*, chloride — *cyan*, water — *pink*, ws-PSCA — *green*) and α4β2-nAChRs in a lipid bilayer (2b–c: DOPC/DOPE/PSM lipids are *grey*, cholesterols are *olive*). **Step 3: Conformational clustering** of the MD trajectories yielded 93 structural clusters for ws-PSCA (3a). For nAchRs, we extracted from two MD trajectories and further processed three dimeric interfaces possibly involved in ligand interactions: β2(+)/β2(−) (53 clusters, 3b), α4(+)/β2(−) (54 clusters, 3c), and β2(+)/α4(−) (57 clusters, 3d). The α4(+)/α4(−) interface was not considered due to results of electrophysiology study. **Step 4: Ensemble docking** is a large series of protein–protein docking runs: 4929 (β2(+)/β2(−)), 5022 (α4(+)/β2(−)), and 5301 (β2(+)/α4(−)), which generated 492900, 502200, and 530100 potential complex configurations, respectively. **Step 5: Non-specific post-scoring stage.** Ensembles of solutions were post-scored by hydrophobic/hydrophilic surface complementarity, buried surface area, and specific ionic and hydrogen bonding patterns. As a result, the solution ensembles were reduced to 5483, 4075, and 1671 structures of complexes for the β2(+)/β2(−), α4(+)/β2(−), and β2(+)/α4(−) interfaces, respectively. **Step 6: Specific post-scoring based on intermolecular contacts frequency analysis.** For the remaining solutions, the recurring nAChR–PSCA interactions (visualized by heatmaps: see Fig. S6) were used for identification of native-like conformations of complexes at β2(+)/β2(−) and α4(+)/β2(−) interfaces; β2(+)/α4(−) interface was regarded as less probable for PSCA binding and not further analyzed. This selection process yielded 14 solutions for the β2(+)/β2(−) interface and 19 solutions for the α4(+)/β2(−) interface. **Step 7: MD of HS α4β2-nAChR/ws-PSCA complexes.** The final structural model of the $(\alpha4)_2(\beta2)_3$-nAChR/ws-PSCA complex was constructed by integrating optimal solutions from the β2(+)/β2(−) and α4(+)/β2(−) interfaces. MD simulation was conducted in a lipid membrane for 500 ns. Lipids are shown in *grey*, cholesterols in *olive*, PSCA in *red*, α4-subunit in *light blue*, β2-subunit in *tan/beige*.

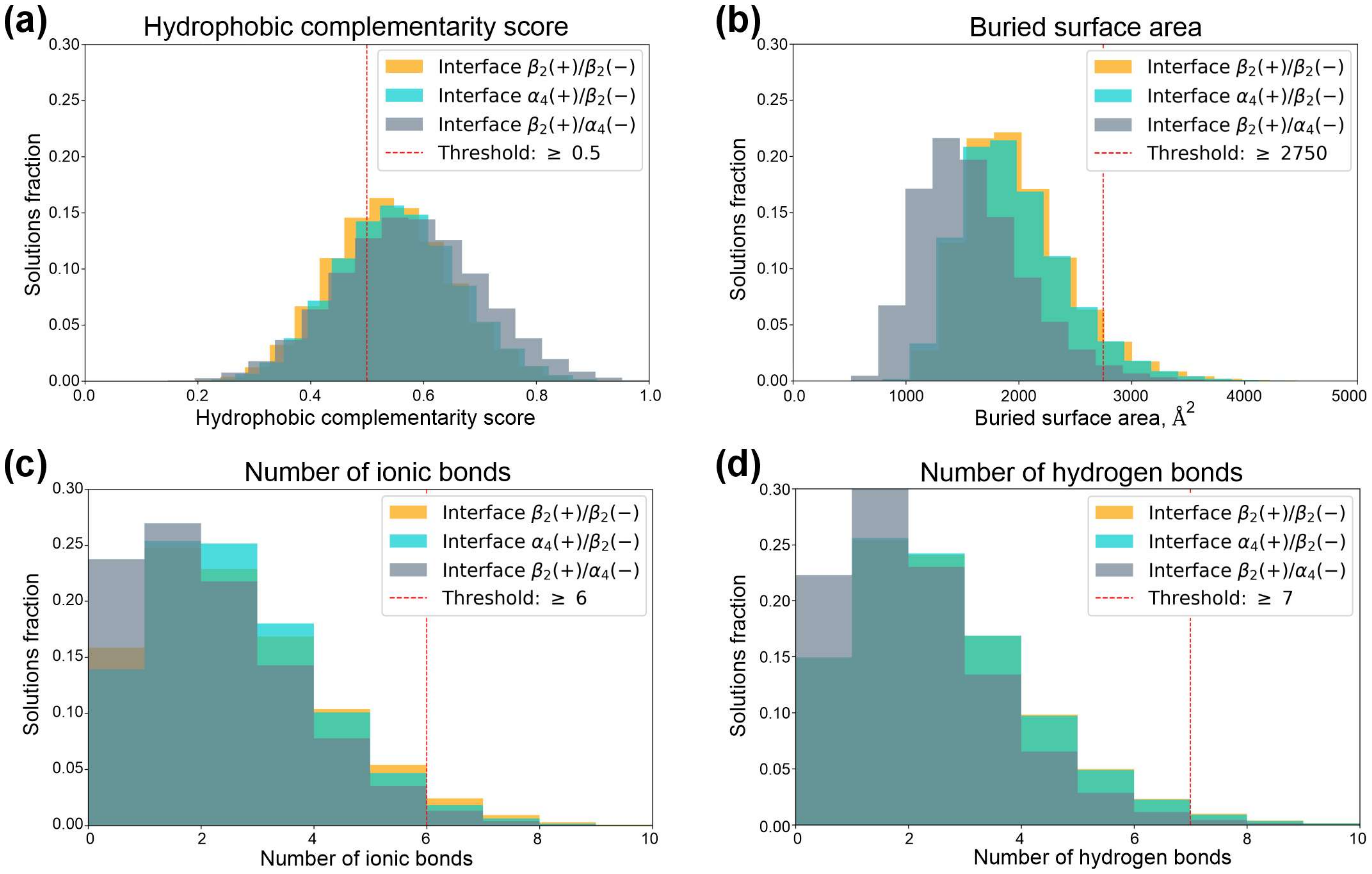

Figure S5. Post-scoring metrics for ws-PSCA docking ensembles into three HS/LS *α*4β2-AChR dimeric interfaces (β2(+)/β2(−): *orange*; *α*4(+)/β2(−): *cyan*; and β2(+)/*α*4(−): *gray*). Figure presents normalized distributions of key post-scoring metrics at its "non-specific" stage. **a:** Hydrophobic complementarity score (fraction of matching hydrophobic/hydrophilic area at the nAChR/ws-PSCA interface). **b:** Buried ws-PSCA surface area. **c, d:** Numbers of intermolecular salt bridges and hydrogen bonds, respectively. *Red dashed lines*: inclusion threshold for each of the four criteria (chosen as ~ 75 % of the maximum value for buried surface area and number of interactions; and 0.5 for hydrophobic complementarity); simultaneous application of all of them reduces initial ~ 500000 ensembles to just 1000–5000 ones. Note that β2(+)/*α*4(−) interface (*gray distributions*) exhibits markedly reduced ionic/hydrogen bonding and buried surface area relative to the two other interfaces, which leads to just ~ 1000 solutions passing this filter for β2(+)/*α*4(−) interface, as compared to ~5K for both β2(+)/β2(−) and *α*4(+)/β2(−) interfaces.

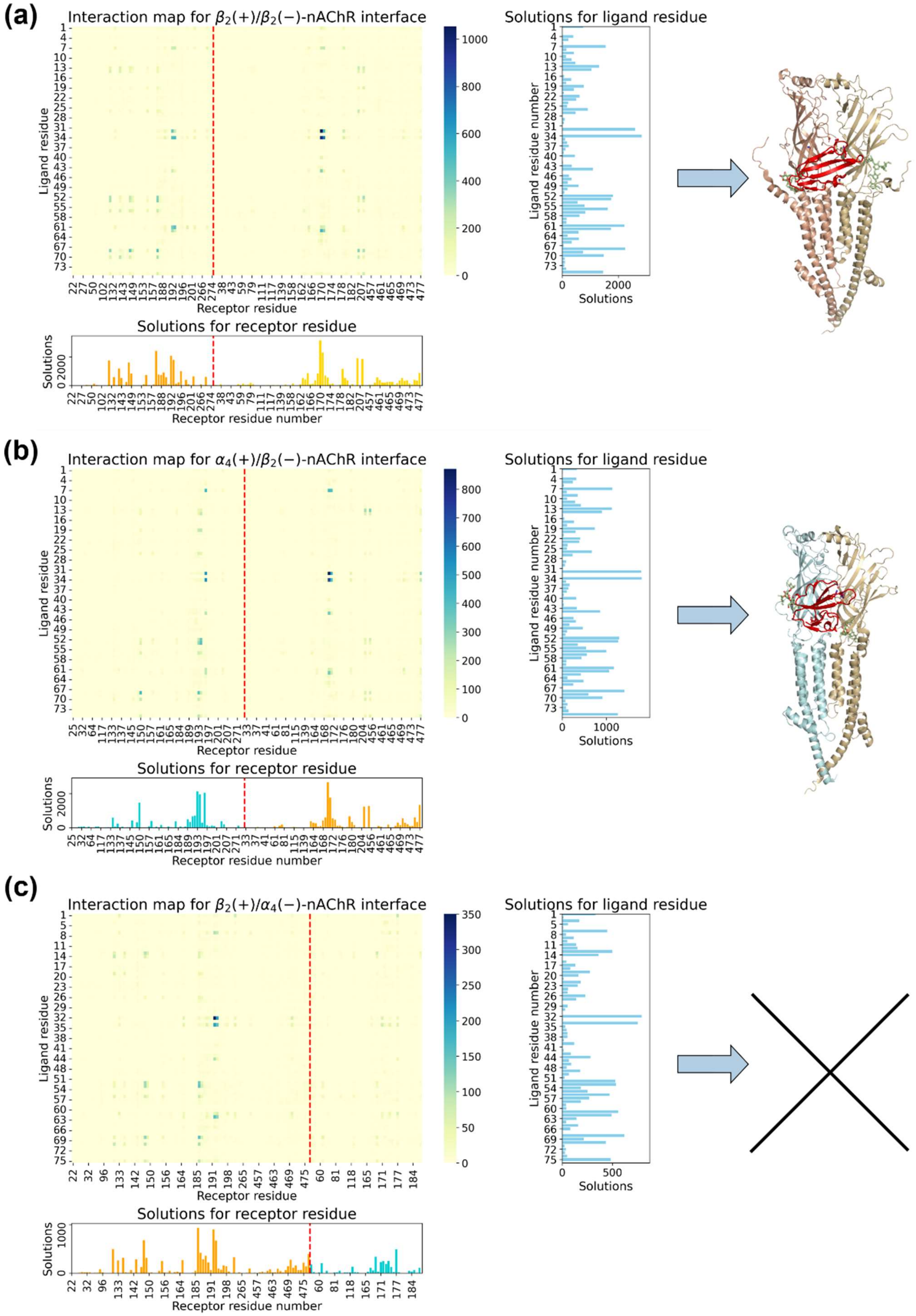
(a)
Interaction map for $\beta_2(+)/\beta_2(-)$-nAChR interface
Ligand residue
Receptor residue
Solutions for receptor residue
Solutions
Receptor residue number
Solutions for ligand residue
Ligand residue number
Solutions
(b)
Interaction map for $\alpha_4(+)/\beta_2(-)$-nAChR interface
Ligand residue
Receptor residue
Solutions for receptor residue
Solutions
Receptor residue number
Solutions for ligand residue
Ligand residue number
Solutions
(c)
Interaction map for $\beta_2(+)/\alpha_4(-)$-nAChR interface
Ligand residue
Receptor residue
Solutions for receptor residue
Solutions
Receptor residue number
Solutions for ligand residue
Ligand residue number
Solutions

Figure S6. Specific post-scoring of nAChR/ws-PSCA ensemble docking, based on intermolecular contacts frequency analysis. Left panels display heatmaps of per-residue interaction frequencies in a non-specifically filtered ensembles for the β2(+)/β2(−) (a), α4(+)/β2(−) (b), and β2(+)/α4(−) (c) interfaces. For each receptor residue–ligand residue pair, the number of docking solutions containing either an ionic or hydrogen bond or stacking interaction between these two residues was calculated. Each heatmap (main field) plots ws-PSCA residue numbers (vertical axis) against nAChR residue numbers (horizontal axis), with a *red dashed line* separating principal (+; left) and complementary (−; right) subunit residues. Interaction frequency is color-coded according to the scale. The bottom bar charts display receptor residue interaction distributions, calculated by summing docking solutions where each receptor residue formed ionic or hydrogen bonds or stacking interactions with the ligand, with subunit-specific coloring (β2: *yellow*; α4: *cyan*). Right-side histograms present equivalent ligand residue data. Note significantly reduced ws-PSCA interaction with α4(−)-subunit, which is in accordance with "netto" worse parameters in Fig. S5 for this interface. Also note that these maps look alike when plotted for the whole (not pre-filtered) ensembles (not shown). Right panel presents final solutions selected through visual inspection after specific post-scoring for β2(+)/β2(−) (a) and α4(+)/β2(−) (b) interfaces (see main text, *Methods* section). For β2(+)/α4(−) (c), we propose that PSCA binding at this interface is unlikely due to rare complementary subunit interactions in the solution ensemble, effectively leaving only principal subunit contacts.

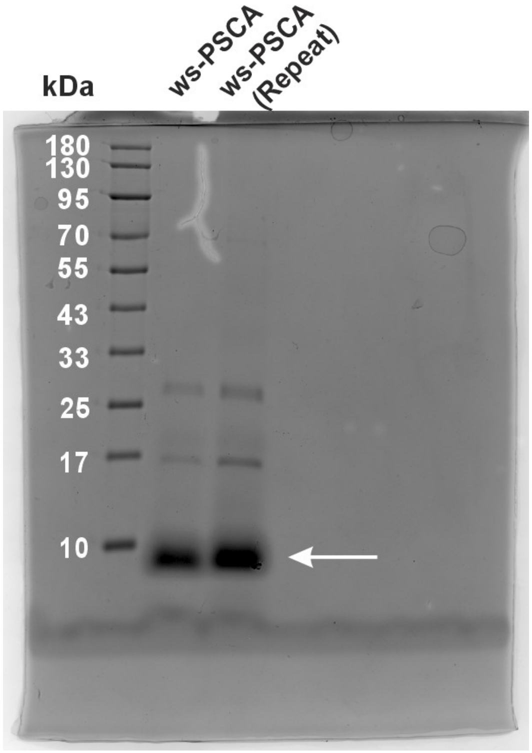

Figure S7. Uncropped and unedited original image of the SDS-PAGE gel of refolded ws-PSCA (MW ~ 8.3 kDa).

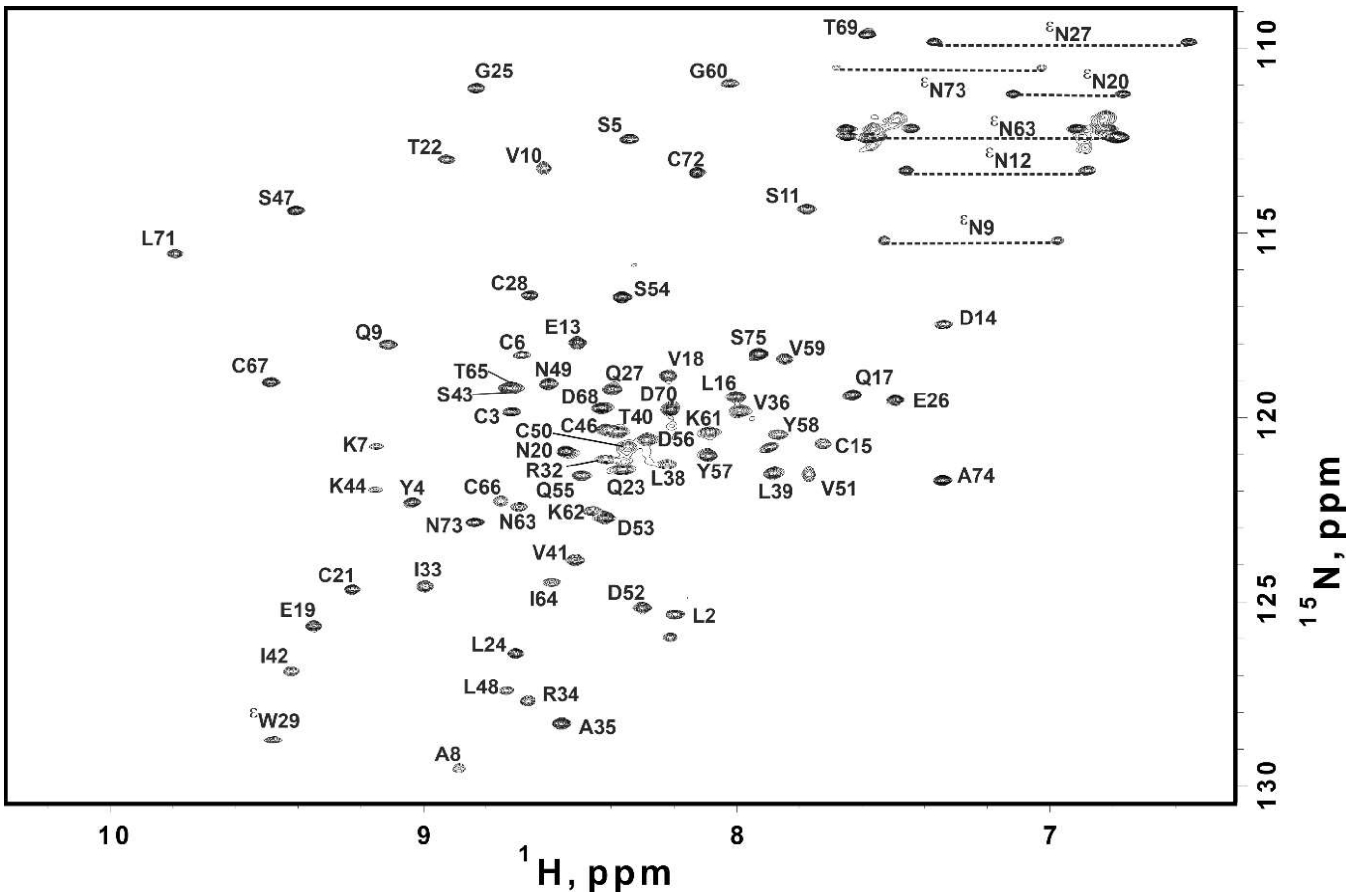

Figure S8. ^{15}N-HSQC NMR spectrum of ^{13}C,^{15}N-labelled ws-PSCA (37°C, pH 7.0, 800 MHz). Assignment of amide groups is shown.

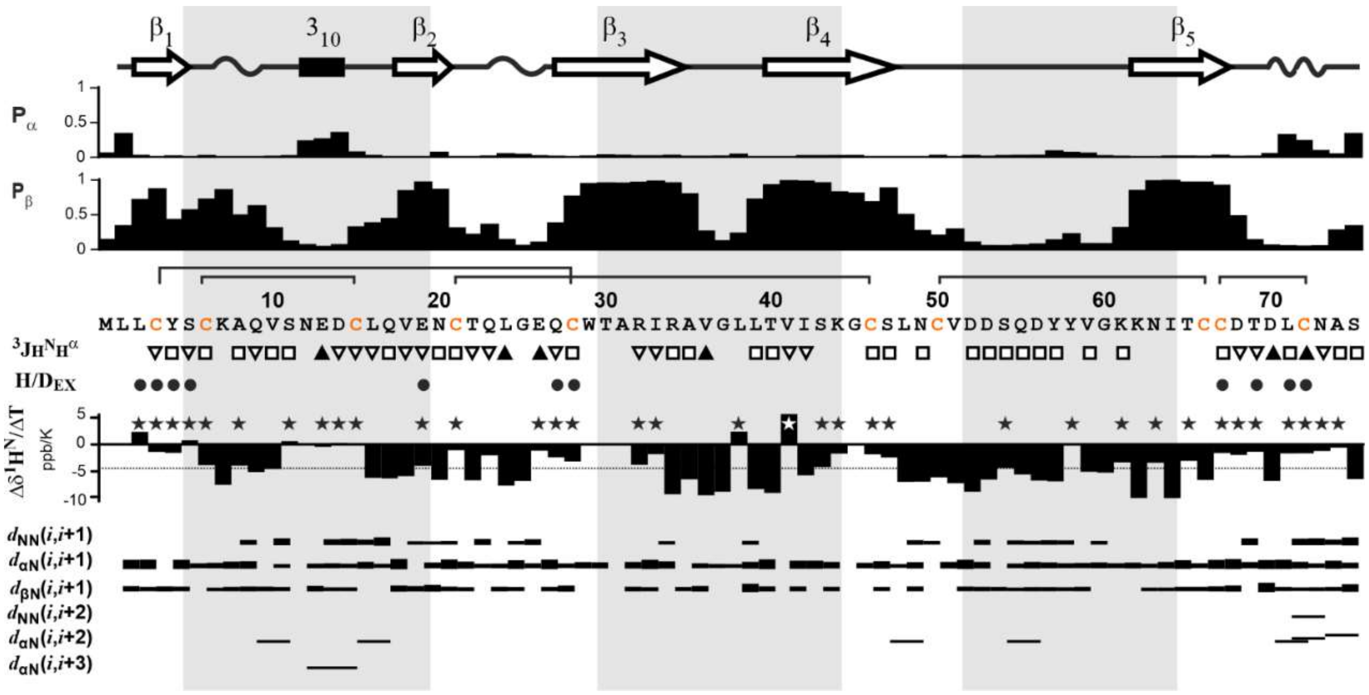

Figure S9. NMR data define secondary structure of ws-PSCA in aqueous solution. P_α and P_β, – Probabilities of α-helix and β-structure formation calculated from ^{1}H, ^{13}C, and ^{15}N chemical shifts in the TALOS-N software. In the sequence Cys residues are highlighted and disulfide connection scheme is shown. $^{3}J_{HNH\alpha}$ - scalar coupling constants between H^N and H^α protons. The small (< 5.5 Hz), large (> 8.5 Hz), and medium (others) couplings are designated by open triangles, filled triangles and open squares, respectively. $\Delta\delta^{1}H^{N}/\Delta T$ – temperature coefficients of amide protons. Amide protons with temperature gradients >-4.5 ppb/K (dotted line) marked by stars. H/D_{EX}, – H-D exchange rates for H^N protons measured at 37°C. The black-filled circles denote H^N protons with relatively large half-exchange time (> 20 min). $\mathbf{d_{xx}}$ - local NOE contacts. Line thickness denotes relative peak intensity.

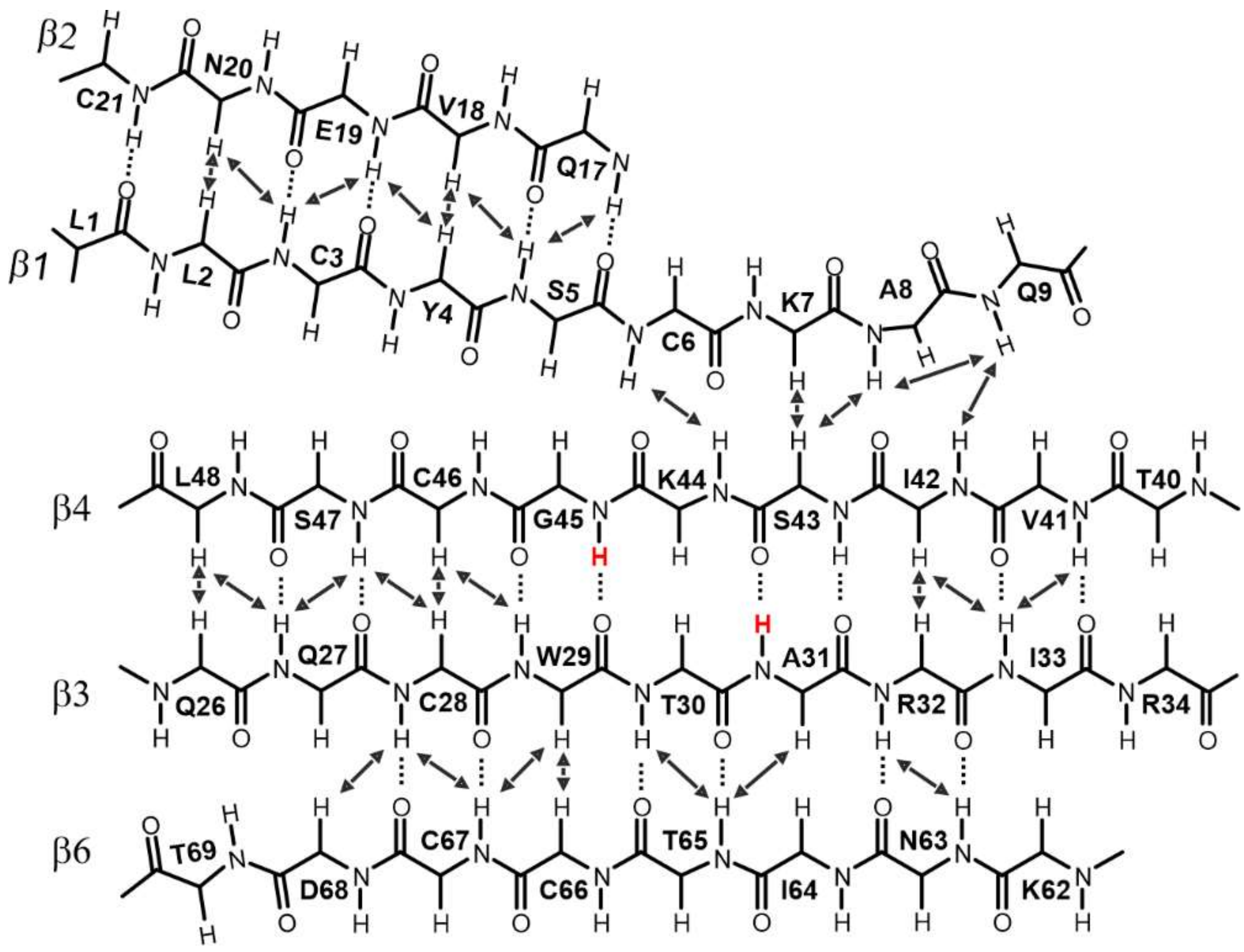

Figure S10. Scheme of contacts between β-strands observed in the NOESY spectra (arrows). Dotted lines denote hydrogen bonds constrains applied for structure calculation. Unobservable in the spectra amide protons (Ala31, Gly45) are marked by red.

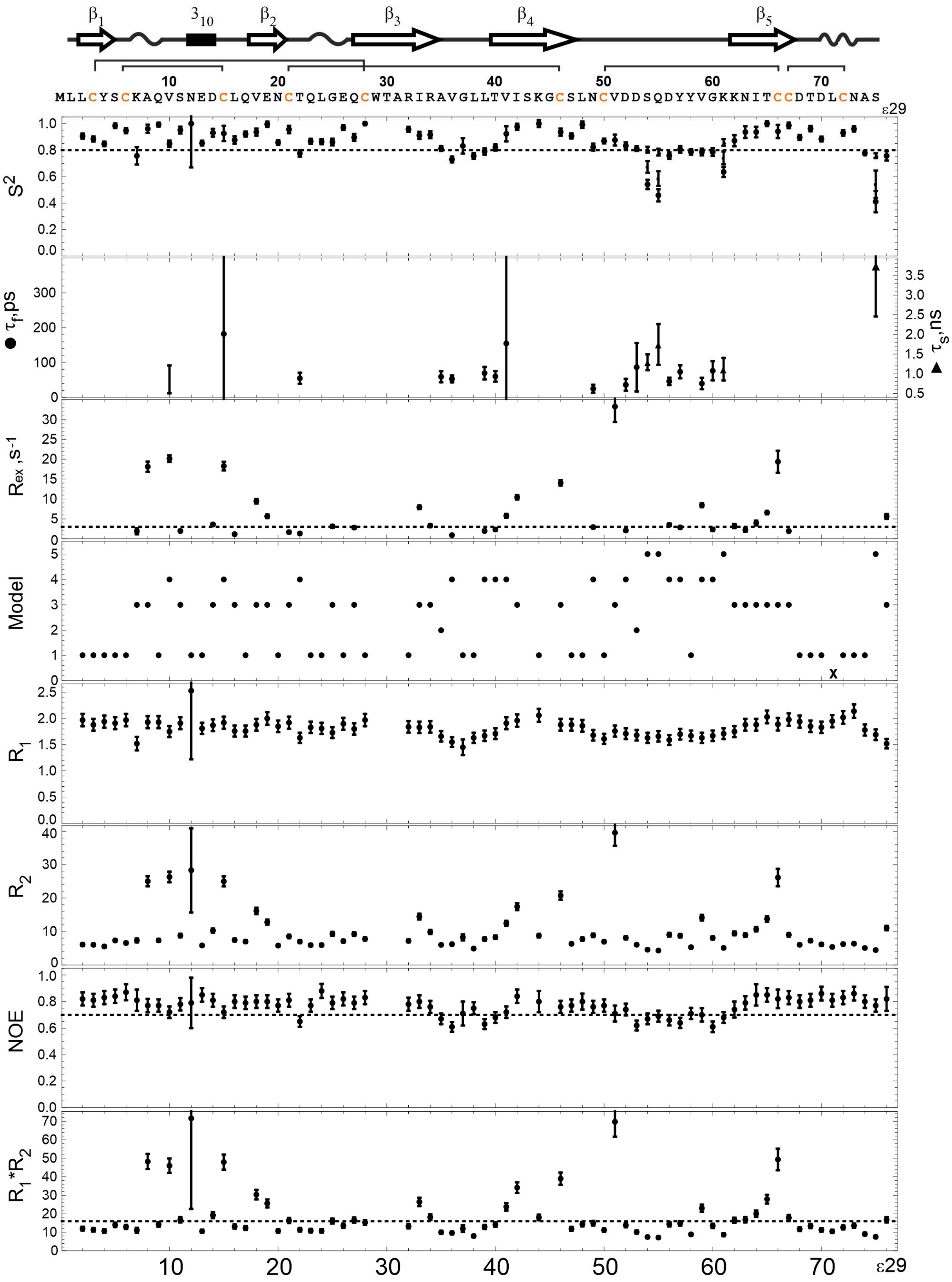

Figure S11. ^{15}N relaxation data and results of the 'model-free' analysis of ws-PSCA (81 MHz, 37°C, pH 7.0) plotted versus sequence. The isotropic overall rotational diffusion model was used. The resulting overall rotational correlation times was 3.8 ns. **Model** – the number of relaxation model assigned by the FastModelFree software. **S^2** – squared values of the generalized order parameter. For model #5 $S^2 = S_f^2 \times S_s^2$, where S_f^2 and S_s^2 are squared order parameters for the fast

and slow motions, respectively. S_f^2 and S_s^2 are shown by squares and triangles, were appropriate. $\boldsymbol{\tau_f}$ and $\boldsymbol{\tau_s}$ – effective correlation times for ps and ns backbone motions, respectively. $\mathbf{R_{ex}}$ – exchange contribution to the transverse relaxation rate. $\mathbf{R_1}$ and $\mathbf{R_2}$ – the values of longitudinal and transverse ^{15}N relaxation rates. **^{15}N-{^{1}H}-NOE** – steady-state heteronuclear NOE. Residues displaying $S^2 < 0.8$ are subjected to extensive motions in ps-ns timescale. Residues displaying $\mathbf{R_1 \cdot R_2} > 16$ s^{-2} or $R_{EX} > 3$ s^{-1} are subjected to exchange fluctuations in μs-s timescale.

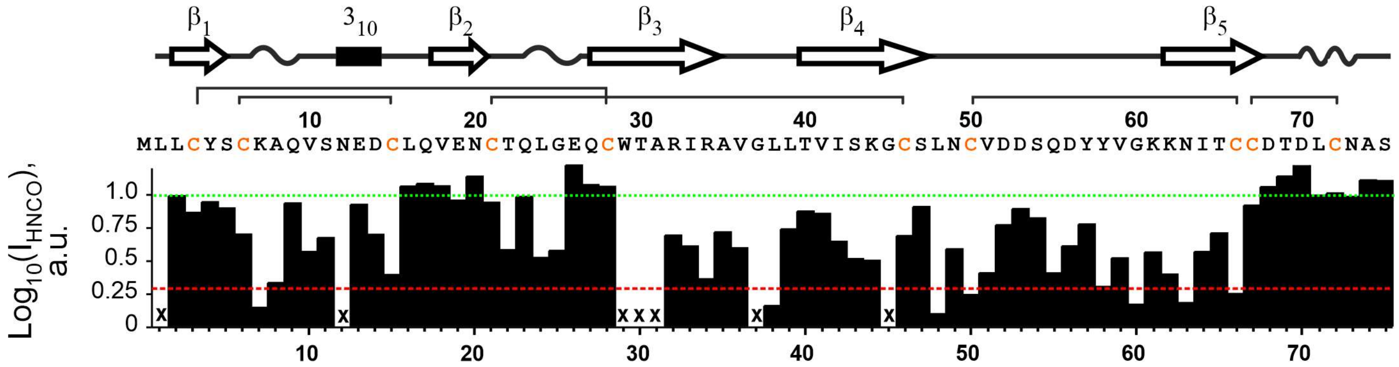

Figure S12. Normalized intensities (Log scale) of signals of ws-PSCA in the 3D HNCO spectrum plotted versus ws-PSCA sequence. Unobservable signals are marked by crosses. Red dashed line indicates the threshold below which the corresponding residue is considered to be involved in slow exchange movements: the signal intensity is 5 times less than the average signal intensity in the "stable" regions of the ws-PSCA back-bone (green dotted line).

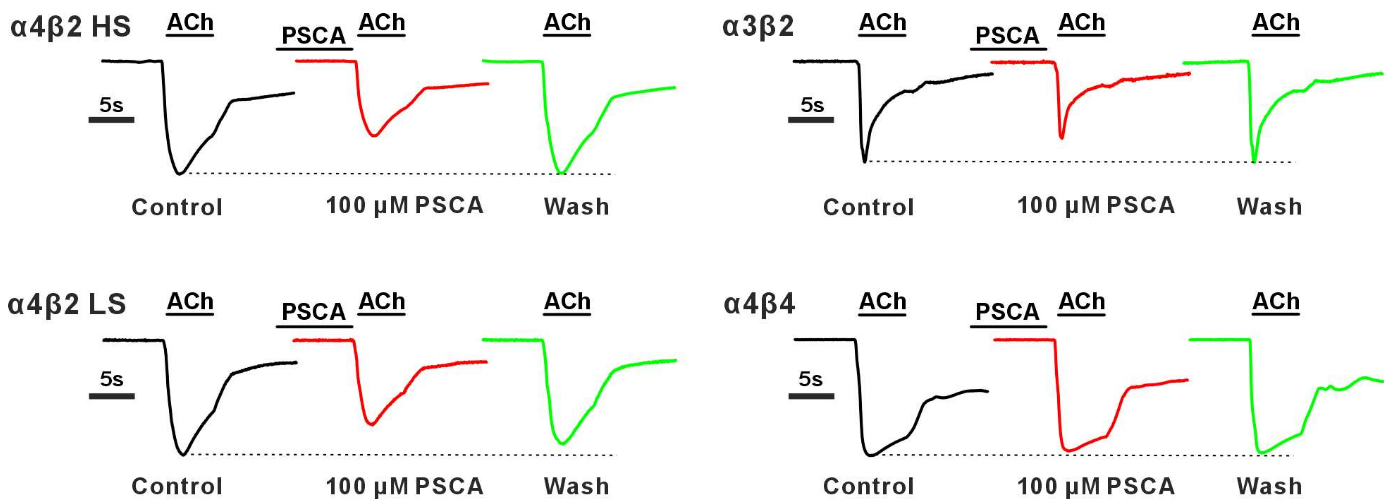

Figure S13. Average currents (n = 7-8 oocytes) evoked by 5 s pulses of ACh at $\alpha 3\beta 2$**,** $\alpha 4\beta 4$**, and** $\alpha 4\beta 2$ **LS/HS nAChRs in the absence (black for control, green for wash) or presence (red) of 100 µM ws-PSCA.** ACh concentrations: 100 µM ($\alpha 3\beta 2$, $\alpha 4\beta 4$, $\alpha 4\beta 2$ LS) and 10 µM ($\alpha 4\beta 2$ HS). Oocytes were pre-incubated with ws-PSCA for 20 s (drawn out of scale).

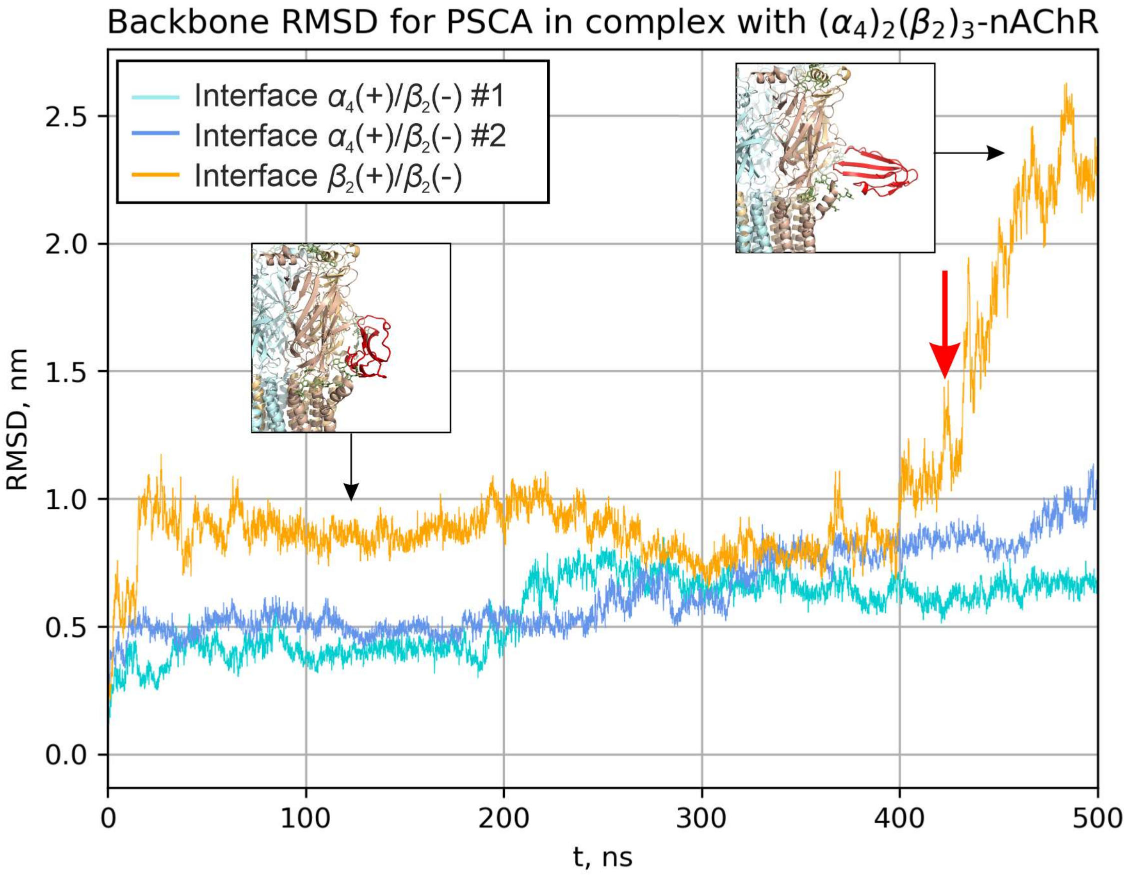

Figure S14. Backbone RMSD of ws-PSCA in complex with HS $(\alpha 4)_2(\beta 2)_3$-nAchR, derived from MD replica #1 (superimposition by receptor backbone). *Orange trace*: ligand RMSD at the β2(+)/β2(−) interface; *blue traces*: α4(+)/β2(−) interfaces. The abrupt deviation in the orange curve at t = 430 ns (indicated by *red arrow*) signifies either initiation of ligand dissociation, or a binding mode transition at the β2(+)/β2(−) interface (illustrated by *insets*). In contrast, both ligand trajectories at $\alpha_4(+)/\beta_2(-)$ interfaces remain stable. In replica #2, ws-PSCA in another interface revealed signs of rearrangement (Fig. S15).

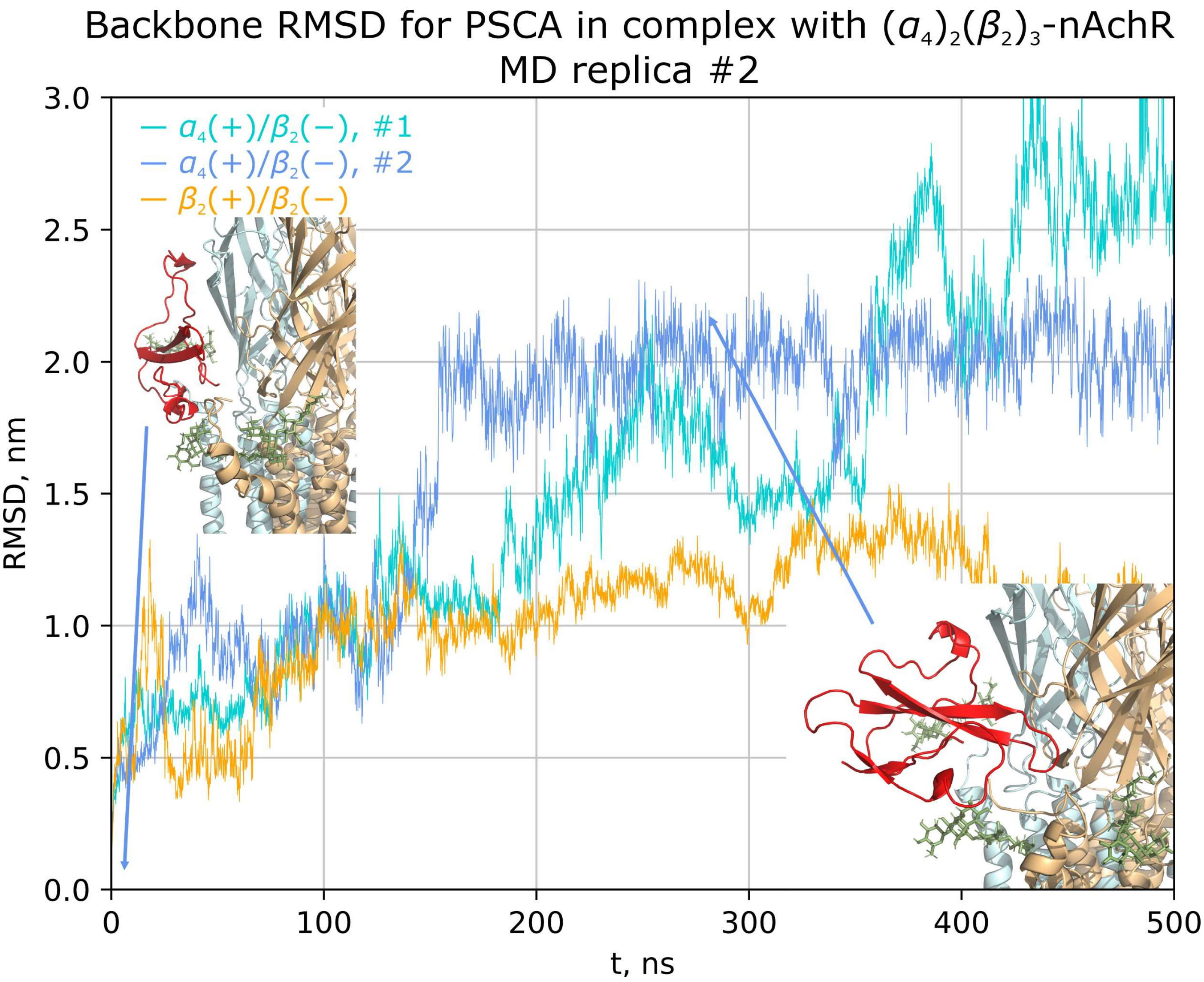

Figure S15. Backbone RMSD of ws-PSCA in complex with HS (α4)$_2$(β2)$_3$-nAChR, derived from MD replica #2 (superimposition by receptor backbone). *Orange trace*: ligand RMSD at the β2(+)/β2(−) interface; *blue traces*: α4(+)/β2(−) interfaces. Contrary to the first simulation (Fig. S13), in this run the PSCA complex was stable at the β2(+)/β2(−) interface, but underwent a conformational rearrangement at the α4(+)/β2(−) interfaces. *Left inset:* Initial conformation at the simulation onset (0 ns). The ligand is bound parallel to the receptor surface. *Right inset:* Snapshot at 280 ns. The ligand has undergone a significant reorientation, positioning its loops toward the receptor interface (similar to β2(+)/β2(−) interface from replica #1, Fig. S14).

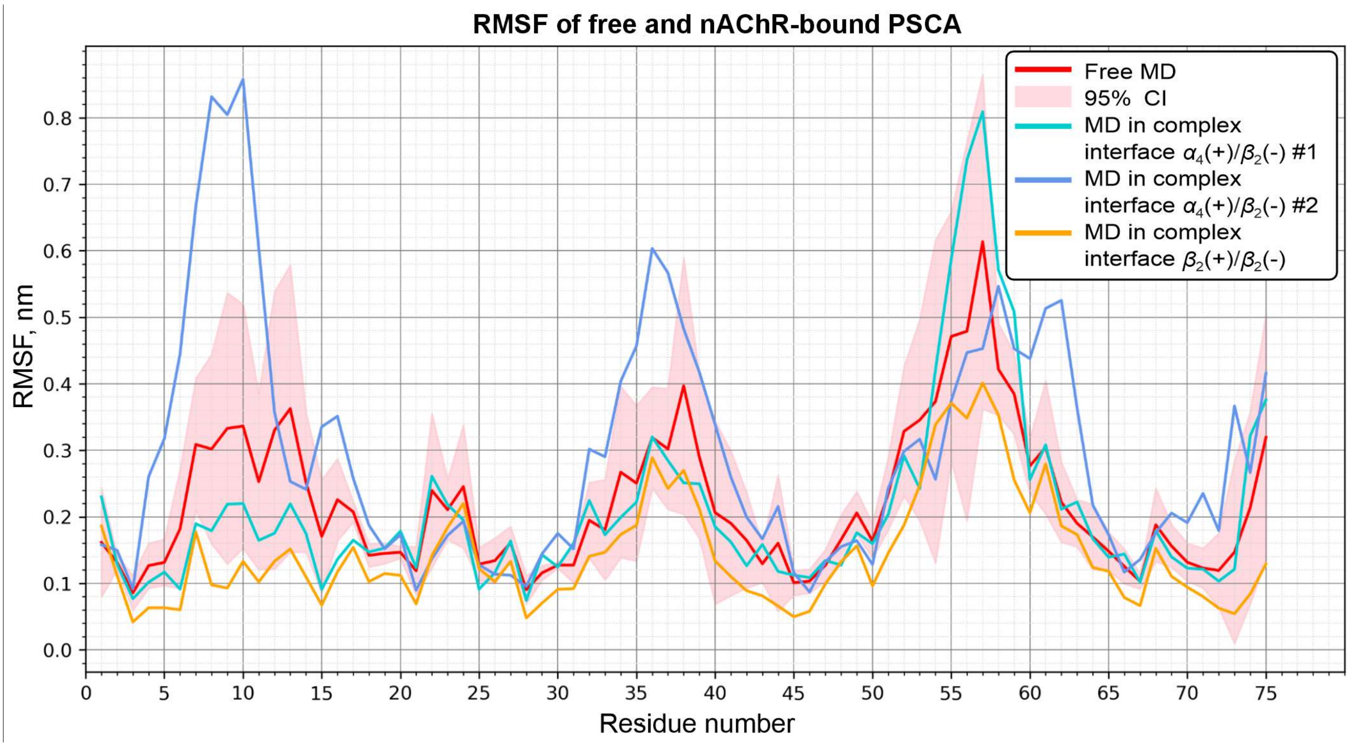

Figure S16. RMSF for free and nAChR-bound ws-PSCA under different conditions: free (*red line* ± *pink fill* for 95% C.I. from several MD trajectories) or bound to nAChR at the β2(+)/β2(−) (*orange*) or α4(+)/β2(−) (*blue* and *cyan*) interfaces in MD replica #1 (Fig. S14). When bound to the α4(+)/β2(−) interfaces, RMSF values for some residues significantly exceed those of the free ligand, while no such increase is observed for the β2(+)/β2(−) interface. This suggests that receptor residues interacting with the ligand at the α4(+)/β2(−) interfaces may induce additional fluctuations in bound PSCA, whereas the β2(+)/β2(−) interface appears more restrictive.

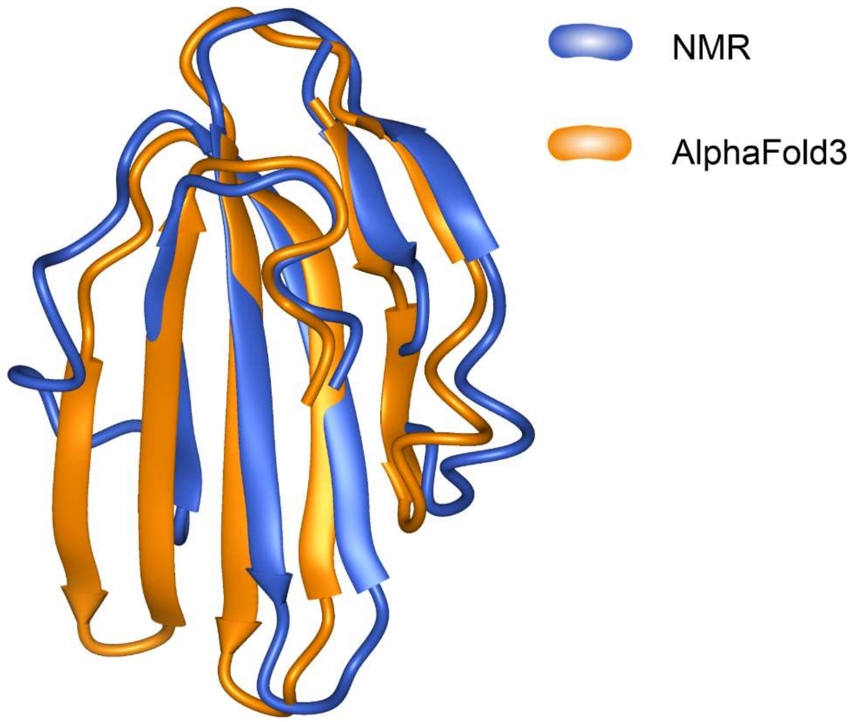

Figure S17. Comparison of the ws-PSCA structures obtained by NMR (blue) and predicted from sequence by AlphaFold3 (orange). Structures are shown in ribbon representation and superimposed by C^{α} atoms of Cys residues.

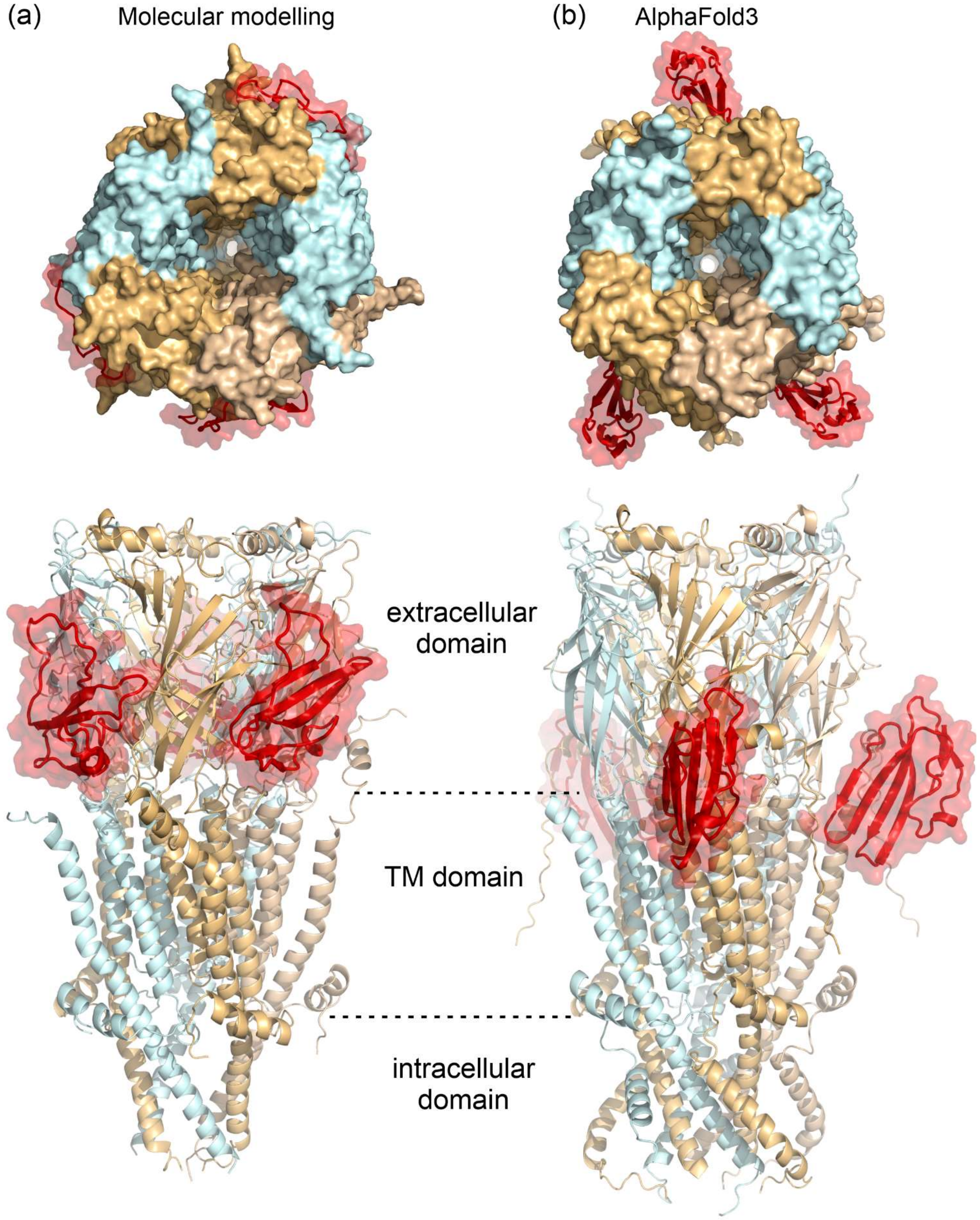

Figure S18. Comparison of the models of the HS (α4)$_2$(β2)$_3$-nAChR in complex with three ws-PSCA molecules obtained by ensemble docking/MD simulations (a; see also Fig. 6 in the main text) and constructed using AlphaFold3 (b). Ws-PSCA is shown in *red*, α4-subunits in *light blue*, β2-subunits in *tan/beige*. Top and side views are shown. The AlphaFold3 software produced unrealistic solutions, the ws-PSCA molecules are bound to the membrane interface of the receptor and the interaction interface in each case involves only one receptor subunit, but not the binding site, which is at the interface between the two subunits.
